# Identification of molecular subtypes and diagnostic model in clear cell renal cell carcinoma based on collagen-related genes may predict the response of immunotherapy

**DOI:** 10.3389/fphar.2024.1325447

**Published:** 2024-02-05

**Authors:** Yulong Hong, Zhengtong Lv, Zhuo Xing, Haozhe Xu, Harripersaud Chand, Jianxi Wang, Yuan Li

**Affiliations:** ^1^ Department of Urology, Second Xiangya Hospital, Central South University, Changsha, Hunan, China; ^2^ Department of Urology, Beijing Hospital, National Center of Gerontology, Institute of Geriatric Medicine, Chinese Academy of Medical Sciences, Beijing, China; ^3^ Department of Urology, New Amsterdam Regional Hospital, New Amsterdam, Guyana; ^4^ Department of Urology, The Third Hospital of Changsha, Changsha, Hunan, China

**Keywords:** clear cell renal cell carcinoma, collagen, molecular subtypes, machine learning, diagnostic model, immunotherapy

## Abstract

**Background:** Collagen represents a prominent constituent of the tumor’s extracellular matrix (ECM). Nonetheless, its correlation with the molecular subtype attributes of clear cell renal cell carcinoma (ccRCC) remains elusive. Our objective is to delineate collagen-associated molecular subtypes and further construct diagnostic model, offering insights conducive to the precise selection of ccRCC patients for immunotherapeutic interventions.

**Methods:** We performed unsupervised non-negative matrix factorization (NMF) analysis on TCGA-KIRC samples, utilizing a set of 33 collagen-related differentially expressed genes (33CRDs) for clustering. Our analysis encompassed evaluations of subtype-associated differences in pathways, immune profiles, and somatic mutations. Through weighted gene co-expression network analysis (WGCNA) and four machine learning algorithms, two core genes were found and a diagnostic model was constructed. This was subsequently validated in a clinical immunotherapy cohort. Single cell sequencing analysis and experiments demonstrated the role of core genes in ccRCC. Finally, we also analyzed the roles of MMP9 and SCGN in pan-cancer.

**Results:** We described two novel collagen related molecular subtypes in ccRCC, designated subtype 1 and subtype 2. Compared with subtype 1, subtype 2 showed more infiltration of immune components, but had a higher TIDE (tumor immunedysfunctionandexclusion) score and increased levels of immune checkpoint molecules. Furthermore, reduced prognosis for subtype 2 was a consistent finding in both high and low mutation load subgroups. MMP9 and SCGN were identified as key genes for distinguishing subtype 1 and subtype 2. The diagnostic model based on them could better distinguish the subtype of patients, and the differentiated patients had different progression free survival (PFS) in the clinical immunotherapy cohort. MMP9 was predominantly expressed in macrophages and has been extensively documented in the literature. Meanwhile, SCGN, which was overexpressed in tumor cells, underwent experimental validation, emphasizing its role in ccRCC. In various cancers, MMP9 and SCGN were associated with immune-related molecules and immune cells.

**Conclusion:** Our study identifies two collagen-related molecular subtypes of ccRCC and constructs a diagnostic model to help select appropriate patients for immunotherapy.

## Background

In recent years, the incidence of kidney cancer, particularly renal cell carcinoma (RCC), has been on the rise (Siegel et al., 2023). RCC is the most prevalent renal malignancy, accounting for approximately 5% of all cancer diagnoses in men and 3% in women (Capitanio et al., 2019). Among the various pathological types, ccRCC is the most common, constituting around 75% of all RCCs and presenting a high mortality rate (Moch et al., 2016). For localized ccRCC, the preferred treatment is surgery; when faced with postoperative recurrence, metastasis, or advanced stages of ccRCC, targeted therapy and immunotherapy are commonly employed (Ljungberg et al., 2022). Despite these treatment modalities, a significant portion of patients do not respond favorably to immune checkpoint blockade (Díaz-Montero et al., 2020). Genomic investigations have unveiled complex heterogeneity within and among tumors in ccRCC patients (Li et al., 2023). To address these challenges, there is an urgent need to enhance our ability to identify high-risk tumor subtypes and discover more effective biomarkers (Barata and Rini, 2017).

Tumor heterogeneity is evident in the intricate tumor microenvironment (Xiao and Yu, 2021). The non-neoplastic ECM significantly influences this environment. Recent research highlights the correlation between ECM composition changes and immunotherapy response (Lim et al., 2019). As a major ECM component, the role of collagen in tumors is gradually being recognized (Necula et al., 2022). Studies demonstrate that oncogenic collagen I homotrimers foster pancreatic cancer cell proliferation, while their deficiency enhances anti-PD-1 immunotherapy efficacy (Chen et al., 2022). Tumor derived type III collagen sustains tumor dormancy, and its disruption restores tumor cell proliferation through DDR1-mediated STAT1 signaling (Di Martino et al., 2022). In breast cancer, collagen promotes tumor growth and invasion through multiple mechanisms (Maller et al., 2021; Li et al., 2023). COL4A1 accelerates liver cancer progression, while XVII collagen drives metabolic reprogramming in lung cancer (Wang et al., 2020; Hsu et al., 2020). In urological tumors, Collagen VI can not only promote the proliferation and invasion of bladder cancer, but also cause integrin α1-deficient CD4^+^ T cells to accumulate in the prostate tumor stroma, thereby inhibiting anti-tumor T cell responses (Piao et al., 2021; Pruitt et al., 2023). However, there is currently an inadequate comprehension of the relationship between collagen and the heterogeneity of the tumor microenvironment in ccRCC.

In this study, we developed a new subtyping system of ccRCC based on prognosis associated collagens. We explored the two new subtypes from multiple perspectives, and based on the core genes, a diagnostic device to distinguish the two subtypes was constructed.

## Methods

### Data collection and sources of data

Collagen related genes (CRGs) were obtained from the Gene Cards (https://www.genecards.org/), and genes with a correlation score greater than 5 were selected (Stelzer et al., 2016). The gene expression RNA-seq count data (535 tumor samples and 72 normal samples), clinicopathological information and CNV (copy number variation) data of TCGA-KIRC were all obtained from the xena website (http://xena.ucsc.edu/) (Goldman et al., 2020). We downloaded the tumor mutation data of TCGA-KIRC using the TCGAbiolinks package (Version 2.27.2). We analyzed the PFS of patients treated with Avelumab + Axitinib in the JAVELIN Renal 101 cohort to evaluate the prognosis of immunotherapy (Motzer et al., 2020).

### Differential analysis

EdgeR package (Version 3.38.4) and Deseq2 package (Version 1.36.0) were used to identify differential expression genes (DEGs) between ccRCC tissue and normal kidney tissue. The identification conditions of DEGs were set as | log2 (fold change) | >2 and *p*-value <0.05. For the differential genes identified between the two kidney cancer subtypes, EdgeR package (Version 3.38.4) and Deseq2 package (Version 1.36.0) were also used for differential analysis, and the standards were also | log2 (fold change) | >2 and *p*-value <0.05. In the analysis among patients of different ages, we defined patients aged 60 and older as elderly patients (Siegel et al., 2018; Motzer et al., 2019). Collagen related DEGs, differential genes for typing, and differential genes between two ccRCC subtypes were visualized with the pheatmap package (Version 1.0.12). We used the tinyarray package (version 2.2.7) to draw the Venn diagram. The ggpubr package (version 0.4.0) was used for the visualization of boxplots after differential analysis, but the difference in mRNA expression of MMP9 and SCGN in ccRCC tissues and normal tissues was analyzed with UALCAN (https://ualcan.path.uab.edu/) (Chandrashekar et al., 2017). The immunohistochemical image data in this study came from the Human Protein Atlas (HPA) database (https://www.proteinatlas.org/) (Sjöstedt et al., 2020).

### Protein-protein interaction (PPI) network construction and correlation analysis

We imported the 33CRDs obtained through univariate cox analysis into the string tool (https://string-db.org/) for PPI network analysis (Szklarczyk et al., 2021). Cytoscape software (version 3.8.2) was used to further analyze the data exported in string for constructing the PPI network. Hub genes and three modules were respectively identified by Cytohubba and MCODE.

### Copy number variation analysis

Using the downloaded ccRCC copy number variation data, we analyzed the frequency of gain or loss of copy number for 33 genes used for disease subtype identification. Afterwards, we visualized the chromosomal loci where copy number variations occurred for these genes using the RCircos package (version1.2.2).

### NMF clustering algorithm was used to cluster the KIRC samples

A NMF clustering algorithm was used to cluster the KIRC samples. When using the NMF algorithm, we chose brunet for clustering. We chose the number of iterations nrun to be 50. The rank was set from 2 to 6 for calculation. Cophenetic was used to determine the optimal number of clusters. The R package Rtsne (version0.16) was used to downscale the samples of subtype 1 and subtype 2, and the downscaling results were visualized with the R packages paletteer (version1.5.0) and ggplot2 (version3.4.0). Verification of clustering stability was completed based on the RECA-EU ICGC cohort.

### Gene set enrichment analysis (GSEA)

The log2FC used in the enrichment analysis was based on the Deseq2 package. Pathways in Kyoto Encyclopedia of Genes and Genomes (KEGG) and Gene Ontology (GO) were taken out for GSEA. The clustetrProfiler package (version4.7.1.3) and the org.Hs.eg.db package (version3.15.0) were used for GSEA, and the enrichplot package (version1.16.2) and the ggplot2 package were used for visualization of the results. The pathway screening criteria were |normalized enrichment score (NES)| > 1, *p*-value <0.05, and pathways meeting these criteria were defined as significantly enriched pathways.

### Immune landscape analysis

We used the estimate package (version1.0.13) to calculate the immune score, stromal score, estimate score, and tumor purity. We used the single-sample GSEA (ssGSEA) algorithm to calculate the active level of immune cells and immune function for each sample. We obtained the TIDE score for each sample at TIDE (http://tide.dfci.harvard.edu/) (Sjöstedt et al., 2020; Fu et al., 2020). We compared the expression of molecules related to immune evasion and T cell exhaustion in subtype 1 and subtype 2, which included PDCD1, TIGIT, LAG3, CTLA4, CD80, and CD86.

### Mutation analysis

The acquisition of TCGA mutation data for ccRCC samples relied on the TCGAbiolinks package (version 2.27.2). We performed mutation analysis on the obtained data by maftools package (version 2.12.0) and then calculated the tumor mutation burden (TMB) for each patient and compared TMB between subtype 1 and subtype 2.

### WGCNA and machine learning model screening for subtype markers

Using the gene expression matrix and subtype grouping information as input data, an appropriate soft threshold β was extracted to construct a co-expression matrix. We set the upper limit of module genes to 6000, set the lower limit of module genes to 30, set the height threshold of module merging to 0.25. Correlation coefficient between the modules and subtypes was calculated. The samples involved in subtypes identification were randomly divided into training set and validation set according to 7:3 using the caret package (version6.0.93). The randomForest package (version 4.7.1.1), kernlab package (version 0.9.32), xgboost package (version 1.7.3.1) and stats package (version 4.2.2) were used to train the four models of RF (random forest), SVM (support vector machine), XGB (extreme gradient boosting) and GLM (generalized linear model) respectively. We visualized the evaluation results through residual reverse cumulative distribution plot (RCDP), boxplot of Residuals (BPR) and gene importance plot. We calculated the receiver operating characteristic (ROC) of the four machine learning models using the pROC package (version1.18.0), and the Area Under the Curve (AUC) value of each model was shown in the legend.

### Build diagnostic models for subtypes

We constructed a diagnostic nomogram with the rms package and drew a calibration curve to represent its calibration. ROC was used to demonstrate the discrimination of the nomogram.

### Single-cell analysis

Single-cell transcriptome sequencing data of KIRC_GSE171306, all from untreated ccRCC samples, were used for analysis. The Tumor Immune Single cell Hub (TISCH) was used for single cell analysis (Sun et al., 2021). The FindMarkers function in the Seurat package was used to calculate DEGs. Subsequently, functional enrichment analysis was performed using ClusterProfiler. Monocle was used to perform pseudotime trajectory analysis (Trapnell et al., 2014).

### Cell culture and transfection

786-O and ACHN cells obtained from Procell Life Science & Technology (Wuhan, China) were used in this study. The shRNAs were purchased from GeneCopoeia (United States). 786-O was cultured in RPMI‐1640 medium containing 10% fetal calf serum (Gibco; United States) and maintained in a humidified atmosphere with 5% CO2 at 37°C. ACHN was cultured in MEM medium containing 10% fetal calf serum (Gibco; United States) and maintained in a humidified atmosphere with 5% CO2 at 37°C. SCGN shRNA or shControl were transfected into 786-O cells and ACHN cells with Lipofectamine 2000 (Thermo Fisher Scientific, United States).

### Quantitative real-time PCR (RT-qPCR)

The RT-qPCR method was reported previously (Ai et al., 2023). RNA was extracted using TRIzol reagent (Thermo Fisher Scientific, United States). RT-qPCR was performed by using a reverse transcription kit and PCR kit (#RR037A PrimeScriptTM RT reagent Kit, #RR430A, TB GreenTM Fast qPCR Mix, Takara Bio Inc. Shigo, Japan) following the manufacturer’s instructions. GAPDH served as the reference gene and the 2^−ΔΔCT^ method was used to quantify fold change. The primer sequences for RT-PCR were provided in Supplementary Figure S1.

### Colony formation assays and transwell assays

Colony formation assays were used to observe cell proliferation ability. The cells counted and diluted were plated on a six-well plate and cultured for 12 days. Next, paraformaldehyde fixation and crystal violet staining were performed. Grouped as follows: 786-O (NC, shSCGN #1, shSCGN #2), ACHN (NC, shSCGN #1, shSCGN #2). According to the same grouping method, we conducted transwell assays to observe the changes of invasion ability. 24-well plates and transwell chambers were used for transwell experiments. Add the serum-free diluted cells to the Transwell chamber (2 × 104 cells per well), add 500 μL 10% FBS culture medium to the well under the chamber, and place it in a 37°C, 5% CO2 incubator for 20 h. The next day, they were fixed with methanol for 30 min and stained with 0.1% crystal violet for 30 min. Finally, the results can be obtained by taking pictures and counting.

### Statistical analysis

We used the Wilcoxon test to determine the difference between the two groups, as well as *p*-value calculations. For survival analysis, the log-rank test and Kaplan-Meier (KM) curve were performed. Univariate Cox regression analysis was used to assess prognostic factors and calculate hazard ratios (HR). The experimental data were presented as the mean ± standard deviation (mean ± SD). GraphPad Prism 5 software was used for calculation of experimental data, Student’s t-test was used to compare values between two groups. One-way analysis of variance (ANOVA) and Tukey’s multiple comparison were used to compare values between more than two groups. Difference was considered statistically significant when the *p*-value was less than 0.05. The significance of the differences was indicated as follows: *p < 0.05; **p < 0.01; ***p < 0.001; not significant, *p* > 0.05.

## Results

### 33CRDs required for subtype identification were found

**FIGURE 1:**
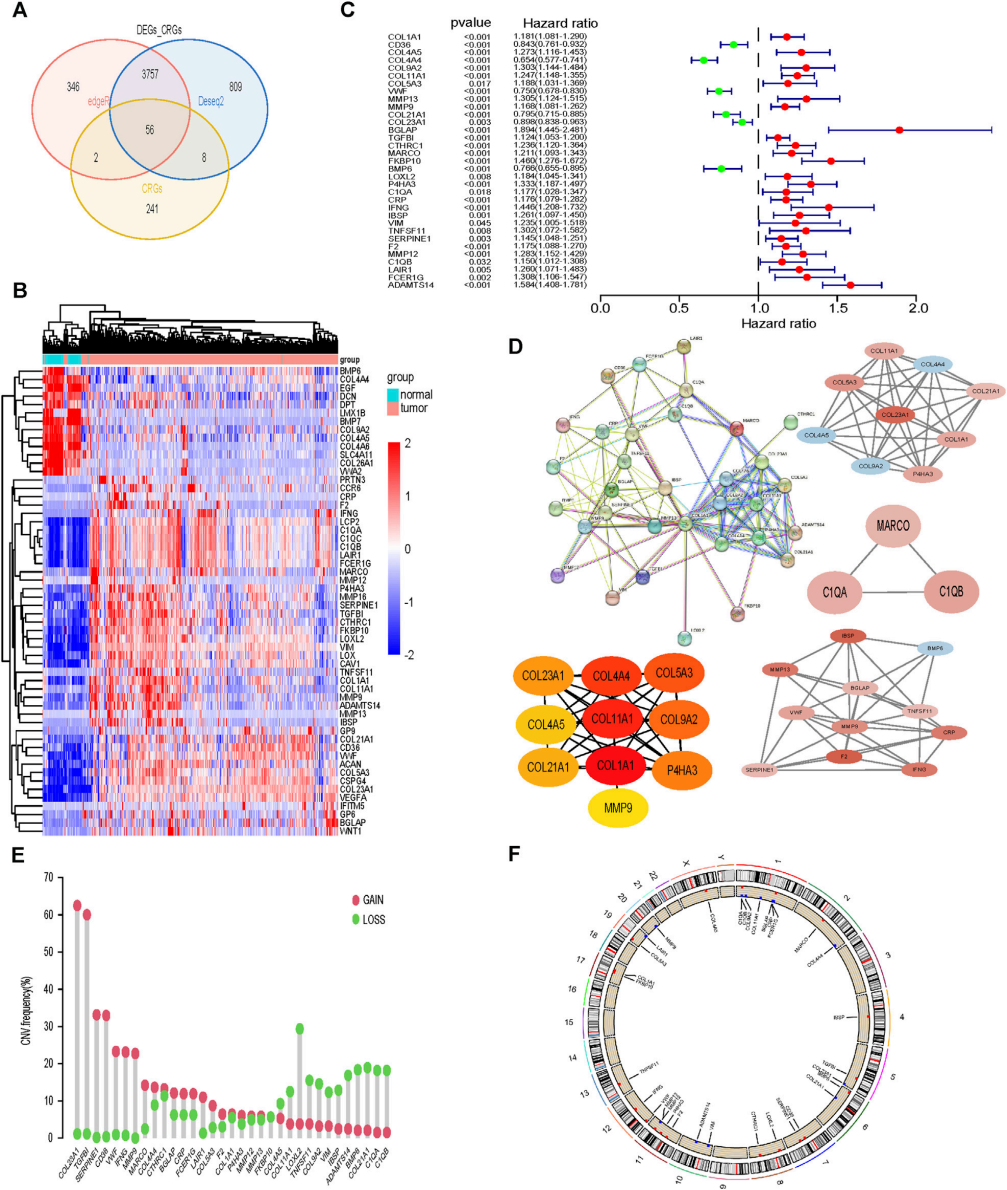
Screening and analysis of 33 CRGs required for subtype identification. **(A, B)** CRGs Differentially expressed between tumor tissues and normal tissues. **(C)** 33CRDs correlated with OS in ccRCC. **(D)** PPI network, core network and core genes of 33CRDs. **(E)** Frequencies of CNV gain, loss, and non-CNV among 33CRDs. **(F)** Circus plots of chromosome distributions of 33CRDs. CRGs, collagen-related genes; 33CRDs, 33 collagen-related DEGs; OS, overall survival; CNV, copy number variation.

Differentially expressed genes between tumor tissue and normal tissue were intersected with 307 collagen-related genes, and finally 56 genes were identified (Figures 1A, B). Subsequently, by univariate Cox analysis, we obtained 33 genes associated with prognosis, among which 6 genes were protective factors and the other 27 genes were risk factors (Figure 1C). The PPI network and hub genes of the 33 genes were shown in Figure 1D. The CNV was common among 33CRDs (Figure 1E). Figure 1F showed the CNV locations on the chromosome for 33 genes.

### Two new collagen-associated subtypes in ccRCC

**FIGURE 2:**
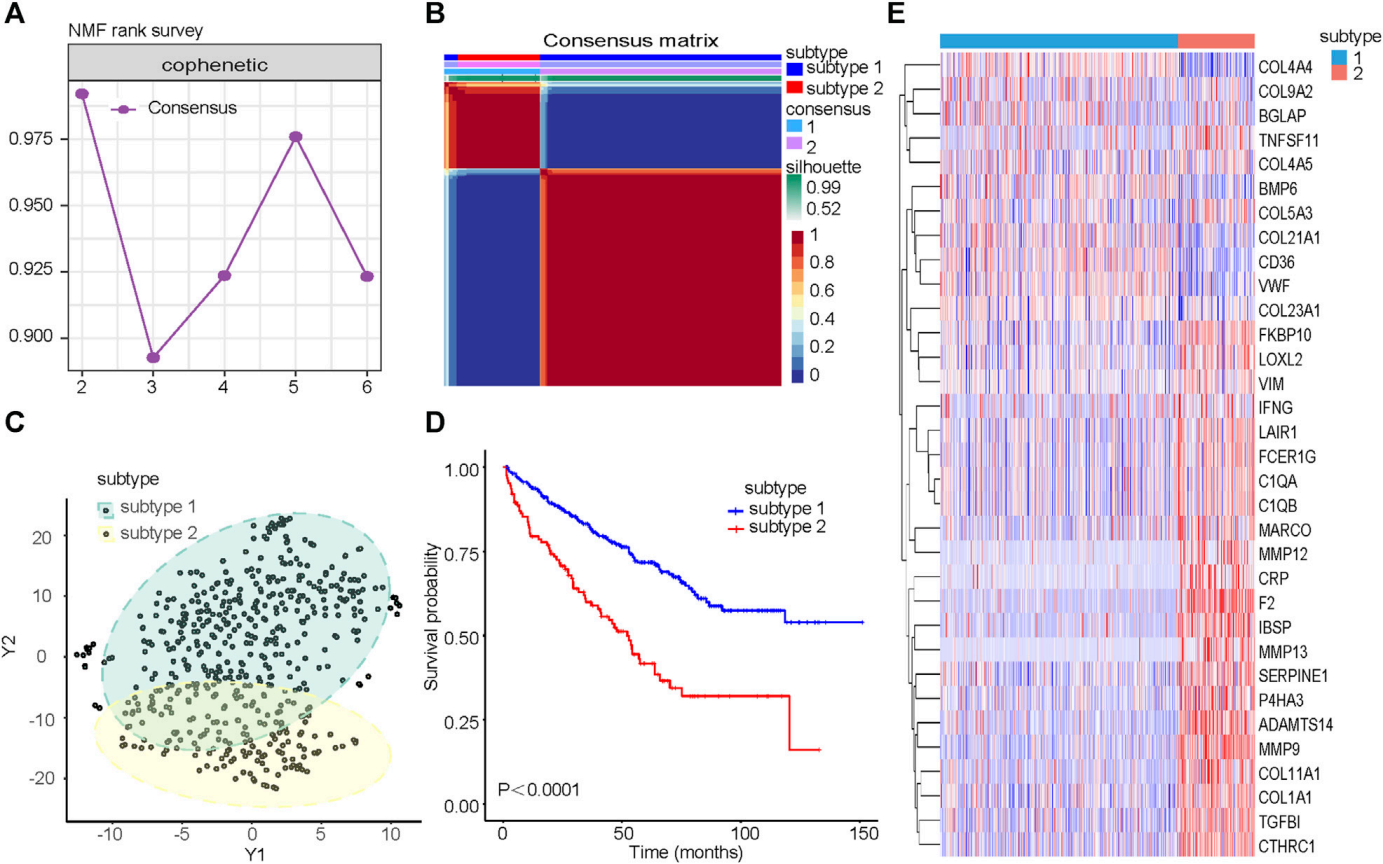
Identification of collagen subtypes in KIRC. **(A)** The cophenetic correlation coefficient is for optimal number of subtypes. **(B)** Consensus matrix of the molecular subtypes: subtype 1 and subtype 2. **(C)** t-SNE scatterplot supports ccRCC subtypes based on mRNA expression profiles. **(D)** Kaplan-Meier OS curves for subtype 1 and subtype 2. **(E)** Expression differences of 33CRDs between subtype 1 and subtype 2.

The cophenetic correlation coefficient was used to determine k, which represented the optimal number of clusters. The optimal number of clusters was determined to be 2 (Figure 2A). We named the two molecular subtypes identified as subtype 1 and subtype 2, as shown in Figure 2B. Subtype 1 and subtype 2 showed significant differences in distribution (Figure 2C) and Overall Survival (OS) (Figure 2D). The expression of the 33CRDs between subtype 1 and subtype 2 was shown in Figure 2E. The clustering result of RECA-EU ICGC samples and 33CRDs showed that they can still be clustered into subtype 1 and subtype 2 (Supplementary Figure S2A). Subtype 1 and subtype 2 showed significant differences in OS (Supplementary Figure S2B) and distribution (Supplementary Figure S2C), which was consistent with the results based on TCGA.

### Functional differences between subtype 1 and subtype 2 in pathways related to immunity and tumor progression

**FIGURE 3:**
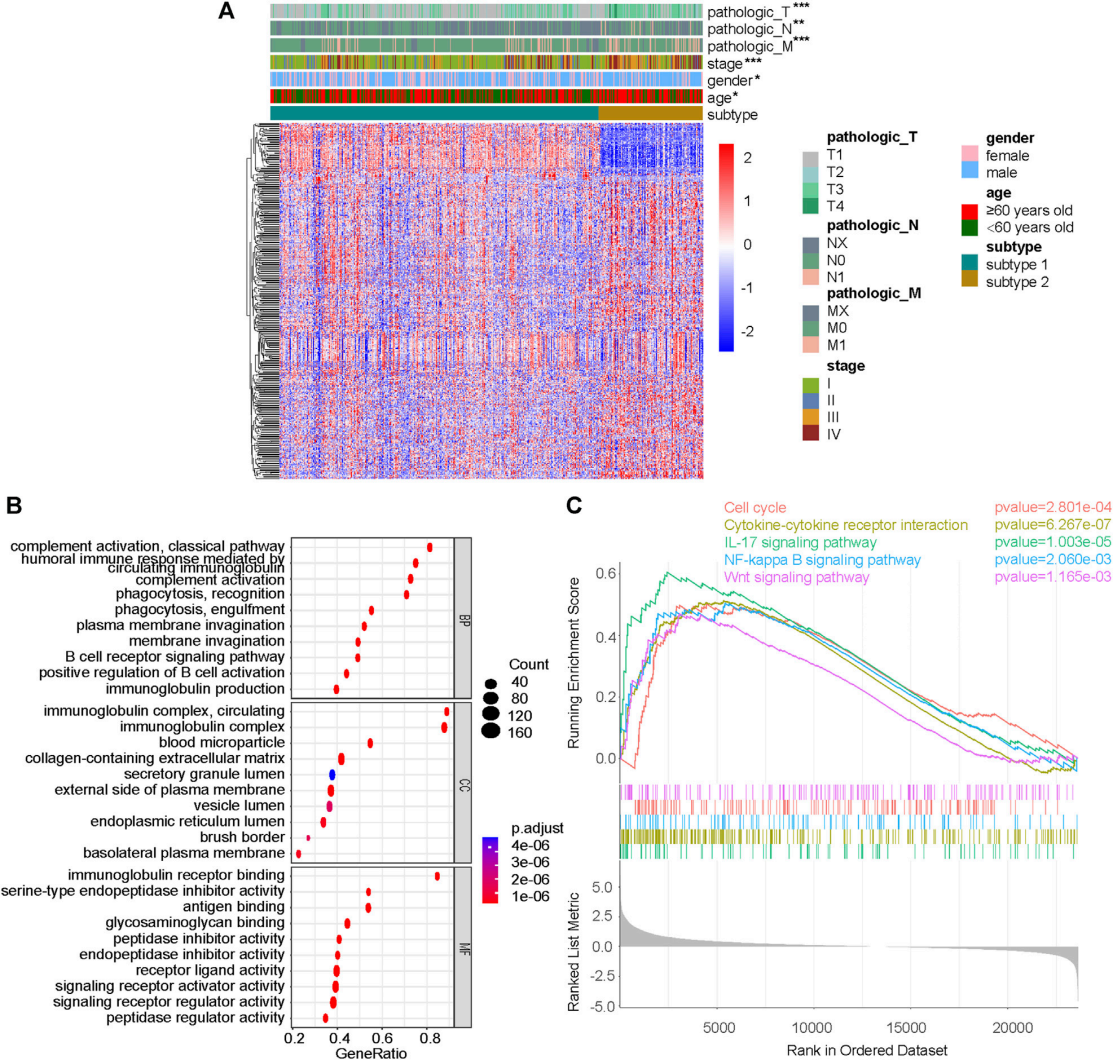
Difference analysis and GSEA between subtype 1 and subtype 2. **(A)** DeSeq2 differential analysis heatmap and corresponding clinical information between subtype 1 and subtype 2. **(B)** GSEA results on pathways in GO, including BP, MF and CC. **(C)** GSEA results on pathways in KEGG. GSEA, gene set enrichment analysis; GO, gene ontology; KEGG, Kyoto-encyclopedia of genes and genomes; BP, biological process; MF, molecular function; CC, cellular component; *p < 0.05; **p < 0.01; ***p < 0.001; not significant, *p* > 0.05.

Differential genes between subtype 1 and subtype 2 were shown together with TNM stage, clinical stage, sex and age (Figure 3A). GSEA was performed on the pathways in GO, and pathways related to immunity and protein secretion were enriched (Figure 3B). GSEA of KEGG related pathways revealed that subtype 1 and subtype 2 mainly differed in Cell cycle, Cytokine-cytokine receptor interaction, IL-17 signaling pathway, NF-kappa B signaling pathway and Wnt signaling pathway (Figure 3C).

### Subtype 1 and subtype 2 had different immune characteristics

**FIGURE 4:**
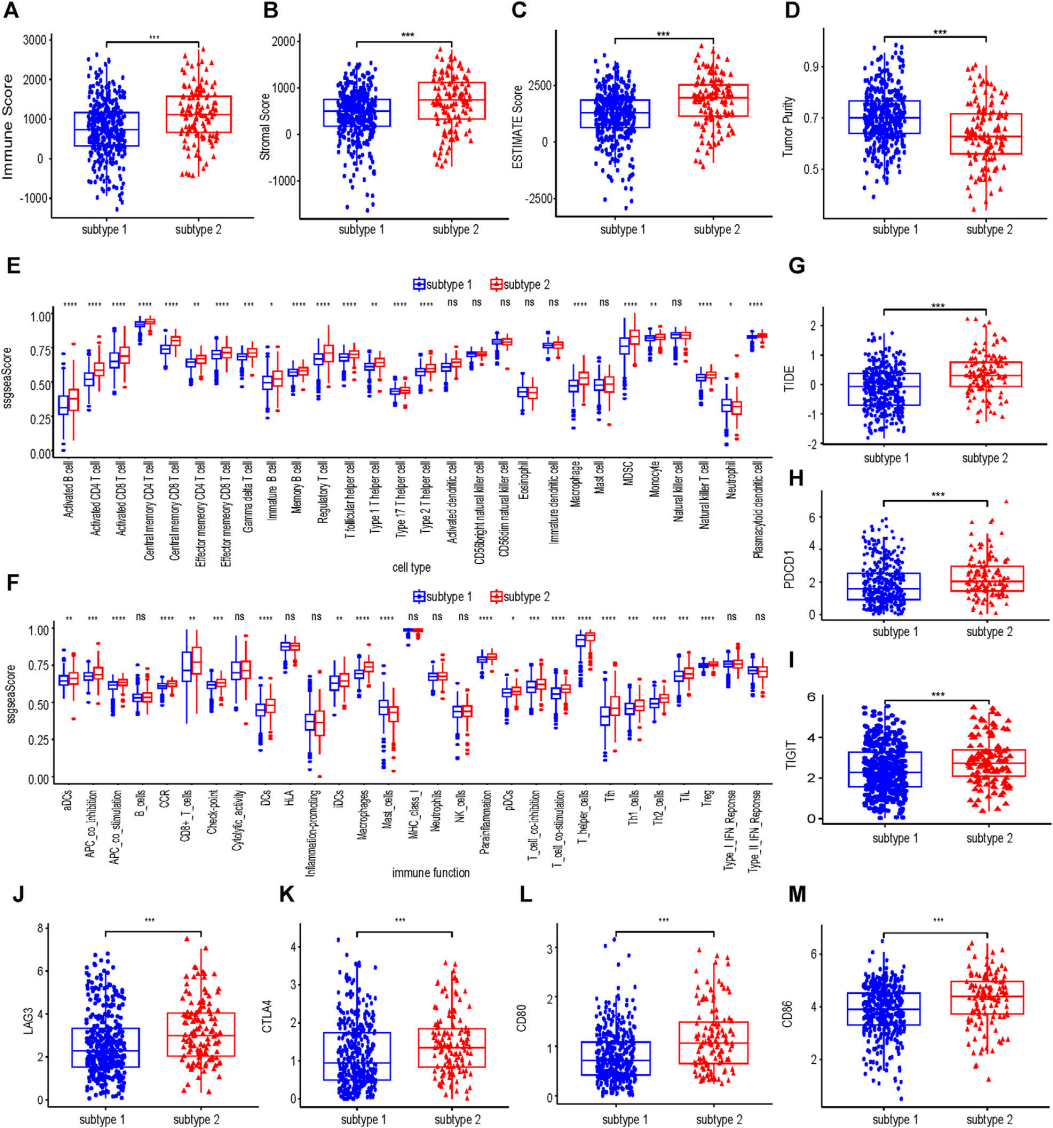
Immune infiltration analysis of collagen-associated ccRCC subtypes. The immune score **(A)**, stromal score **(B)**, ESTIMATE score **(C)** and tumor purity **(D)** between subtype 1 and subtype 2. Comparisons of immune cells **(E)** and immune functions **(F)** between subtype 1 and subtype 2. **(G)** The differences in the TIDE score between subtype 1 and subtype 2. **(H–M)** Differences in expression of six molecules related to Immune evasion and T cell exhaustion compared between subtype 1 and subtype 2. TIDE, tumor immunedysfunctionandexclusion.

As shown in Figures 4A–D, the immune score, stromal score and estimate score of subtype 2 were higher than those of subtype 1, but the tumor purity of subtype 2 was lower than that of subtype 1. Subtype 2 was associated with more immune cell infiltration (Figure 4E). In terms of immune function, most of the immune functions of subtype 2 were stronger than those of subtype 1 (Figure 4F). TIDE analysis showed that the TIDE score of subtype 2 was significantly higher than that of subtype 1 (Figure 4G), predicting that subtype 2 had poorer immunotherapy efficacy. Compared with subtype 1, subtype 2 had higher expression of PDCD1, TIGIT, LAG3, CTLA4, CD80, and CD86 (Figure 4H–L).

### Tumor mutation characteristics of subtype 1 and subtype 2

**FIGURE 5:**
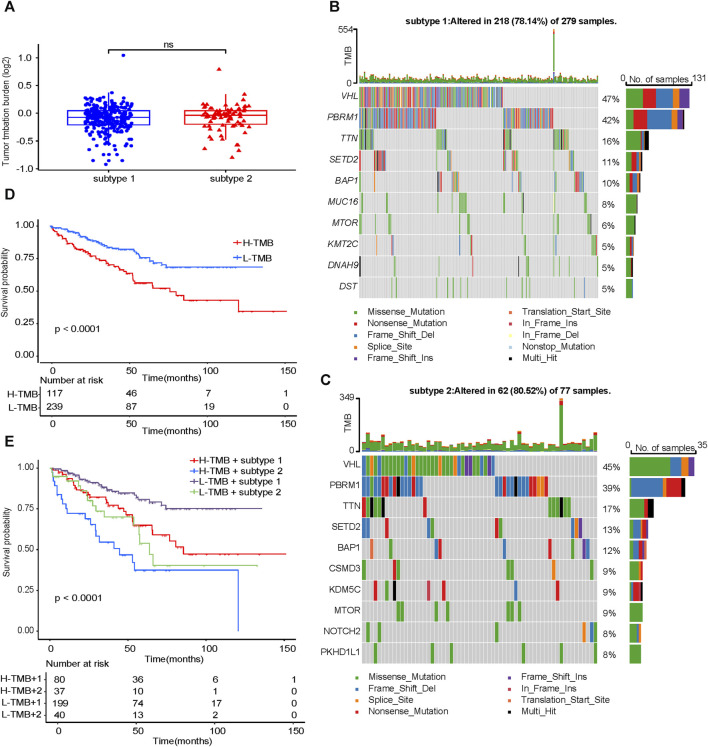
Analysis of TMB characteristics. **(A)** Comparison of TMB between subtype 1 and subtype 2. Waterfall maps of the somatic mutations in the subtype 1 **(B)** and the subtype 2 **(C)**. **(D)** Difference in OS between high TMB and low TMB groups. **(E)** Difference in OS based on TMB and two subtypes. TMB, tumor mutation burden. OS, overall survival.

TMB did not differ significantly between subtype 1 and subtype 2 (Figure 5A). Compared with subtype 1, in subtype 2, VHL had a higher proportion of missense mutation, and PBRM1 had a higher proportion of frameshift deletion (Figures 5B, C). The prognosis of the low TMB group was significantly better than that of the high TMB group (Figure 5D). Combined with the identified two subtypes of ccRCC (Figure 5E), it can be concluded that the prognosis of subtype 2 was worse than that of subtype 1 no matter in the high—TMB group or the low - TMB group. Moreover, the prognosis of high - TMB + subtype 2 was significantly worse than that of low - TMB + subtype 1.

### MMP9 and SCGN were screened as core gene markers of two ccRCC subtypes

**FIGURE 6:**
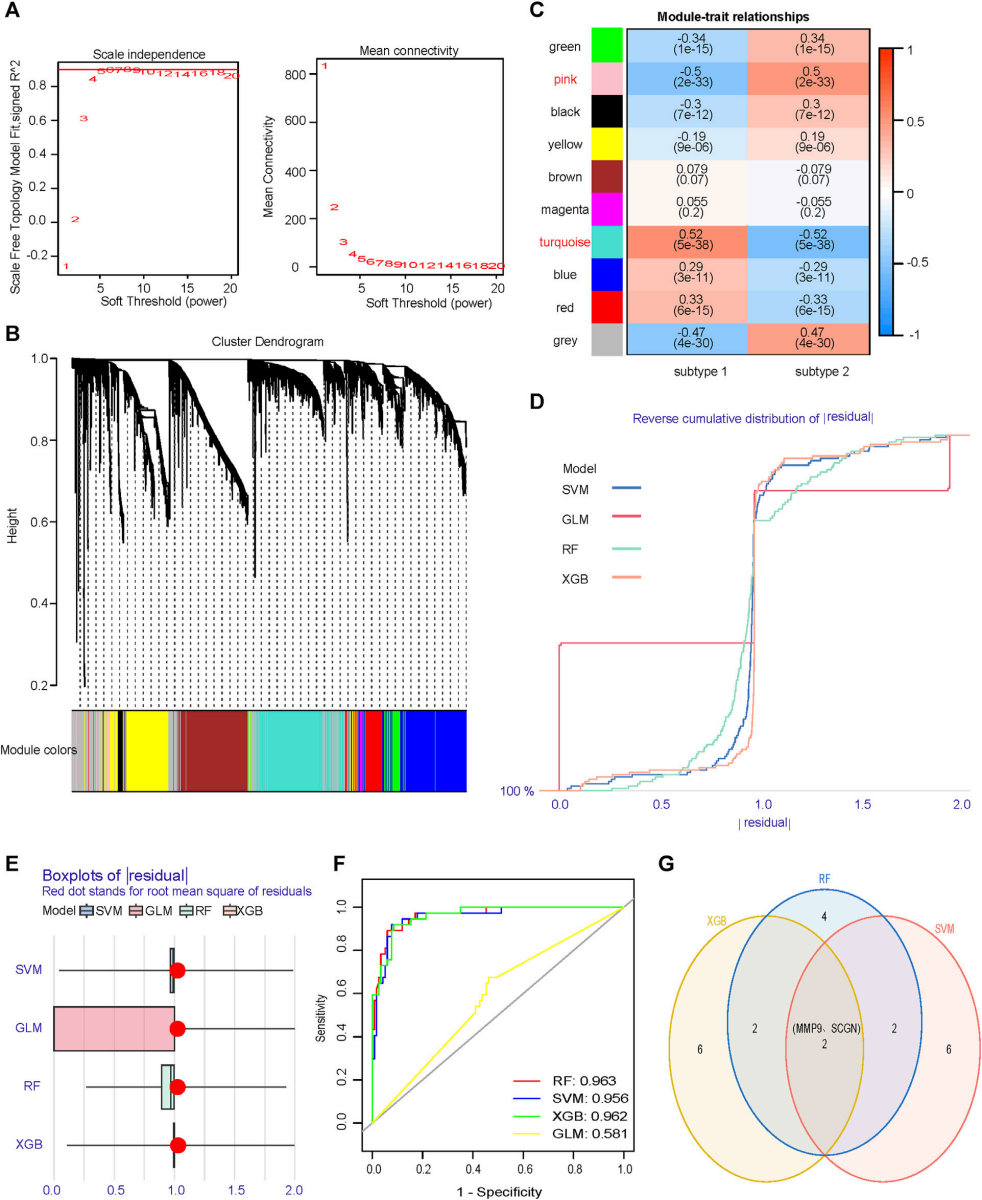
Identification of core genes that differentiate subtypes. **(A)** Scale independence and mean connectivity analyzes are used to determine the optimal soft threshold. **(B)** Gene dendrogram as a result of clustering, where colored rows below the dendrogram indicate different modules. **(C)** Heatmap of correlations between modules and two subtypes. **(D)** RDCP for RF, SVM, XGB, and GLM, each curve represents a model. **(E)** BPR for RF, SVM, XGB, and GLM, each boxplot represents a model. **(F)** ROC represents the discriminative performance of the four machine learning models for subtype 1 and subtype 2 in the validation set. **(G)** The most important top ten genes of the three models with significantly high and similar predictive performance are intersected. RDCP, reverse cumulative distribution plot; RF, random forest; SVM, support vector machine; XGB, extreme gradient boosting; GLM, generalized linear model; BPR, boxplot of Residuals; ROC, receiver operating characteristic.

No outliers were detected during sample clustering. A minimum soft threshold value of 5 for building a scale-free network was finally extracted (Figure 6A). We prohibited gene redistribution within modules, and constructed a co-expression network. A dendrogram (Figure 6B) containing the module colors was drawn to show the module division results of the gene co-expression network. The modules (pink module and turquoise module) with |correlation coefficients|≥0.5 were selected for further analysis (Figure 6C). We built machine learning models using the training set data and validated its performance in the validation set. When |residual|≤1, the curves of RF, SVM and XGB closed to 100%, almost all observations were covered, and the prediction accuracy of the models were high (Figure 6D). In BPR, RF, SVM and XGB had smaller box ranges which indicated better predictive performance (Figure 6E). The AUCs of RF, SVM, XGB and GLM were 0.963, 0.956, 0.962, and 0.581, respectively (Figure 6F). We selected the top 10 most important genes in each model (Supplementary Figure S3). Taking the intersection of the genes selected from the three models with the best performance, it was found that MMP9 and SCGN were the genes they shared (Figure 6G).

### Diagnostic nomogram could distinguish patients receiving immunotherapy into subtype 1 and subtype 2, whose PFS were different

**FIGURE 7:**
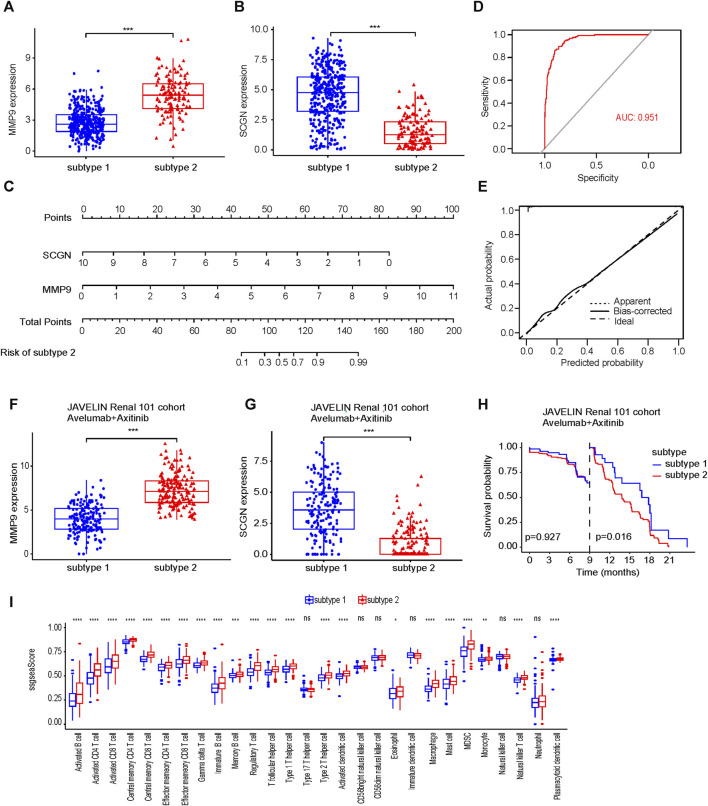
Diagnostic model based on MMP9 and SCGN can predict immunotherapy efficacy. **(A, B)** The expression level of MMP9 and SCGN in subtype 1 and subtype 2. **(C)** Nomogram of diagnostic model. **(D)** ROC of nomogram. **(E)** Calibration curve of nomogram. **(F, G)** In the JAVELIN Renal 101 cohort, the expression level of MMP9 and SCGN in subtype 1 and subtype 2. **(H)** In the JAVELIN Renal 101 cohort, patients treated with avelumab + axitinib are classified as having low PFS in subtype 2. PFS, progression free survival. **(I)** Immune cell infiltration of subtype 1 and subtype 2 in the immunotherapy cohort.

The expression level of MMP9 in subtype 2 was higher than that in subtype 1, and the expression level of SCGN in subtype 2 was lower than that in subtype 1 (Figures 7A, B). We quantified the magnitude of the molecular changes and found that the magnitude of changes was greater in SCGN than in MMP9 (Supplementary Figure S4A). We constructed a diagnostic nomogram based on the expression of MMP9 and SCGN to distinguish subtypes (Figure 7C). The AUC of this nomogram was 0.951 (Figure 7D). The calibration curve indicated good calibration (Figure 7E). Patients treated with avelumab + axitinib in the JAVELIN Renal 101 cohort were distinguished by our nomogram into subtype 1 and subtype 2. The expression level of MMP9 was increased in subtype 2, while the expression level of SCGN was increased in subtype 1 (Figures 7F, G). We quantified the magnitude for changes of molecules (Supplementary Figure S4B). There was a significant difference in prognosis between subtype 1 and subtype 2 starting at 9 months of treatment. (Figure 7H). In the immunotherapy cohort, immune cell infiltration was similar to that in TCGA (Figure 7I).

### Single cell distribution characteristics of MMP9 and SCGN were different

**FIGURE 8:**
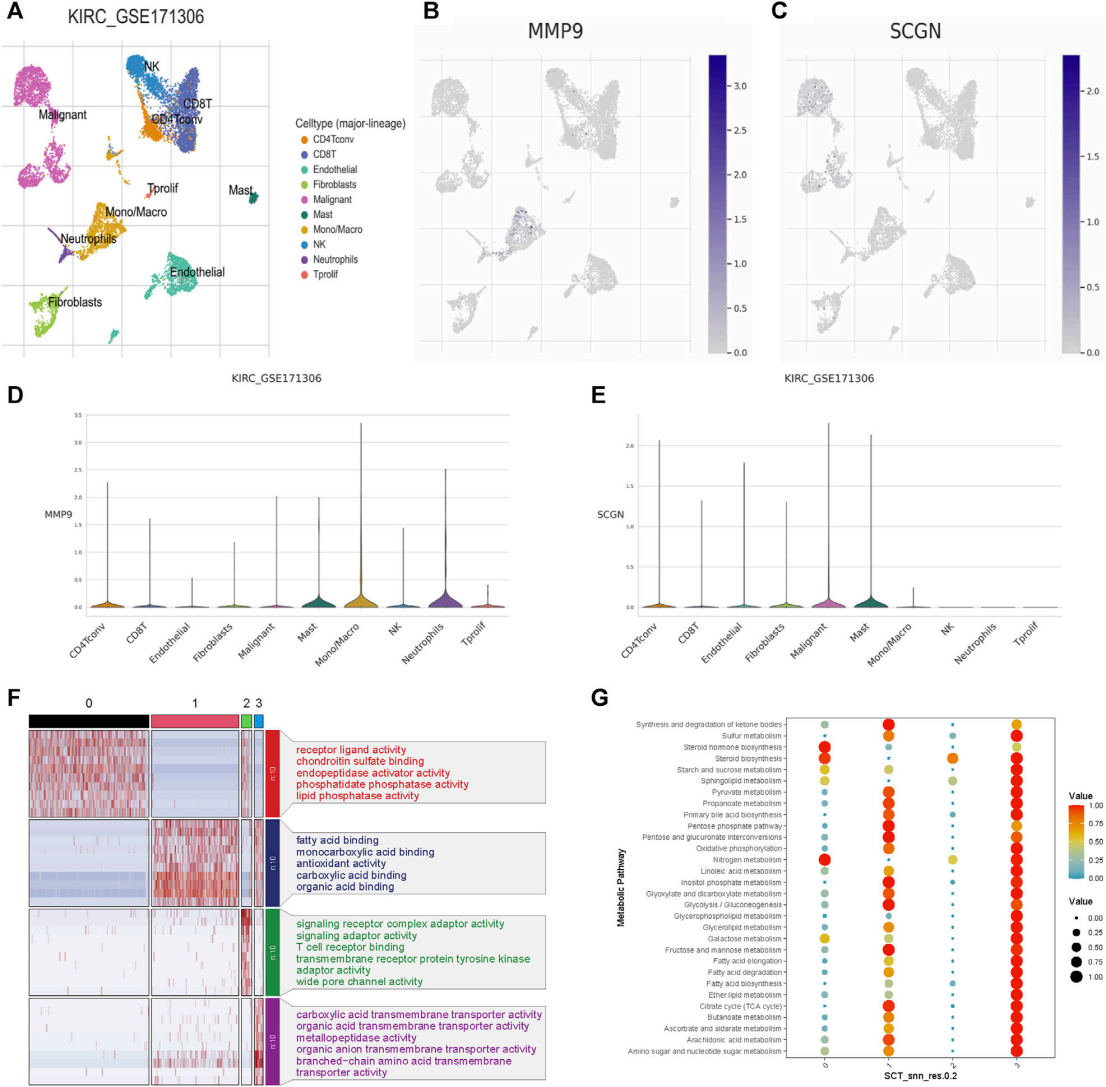
Single-cell expression analysis of MMP9 and SCGN in ccRCC. **(A)** Cell clustering of GSE171306. **(B)** Distribution of MMP9 in different cell populations. **(C)** Distribution of SCGN in different cell populations. **(D)** Expression levels of MMP9 in different cell populations. **(E)** Expression levels of SCGN in different cell populations. **(F)** Molecular function enrichment analysis of SCGN in different subpopulations of malignant cells. **(G)** Enrichment analysis of metabolic pathways of SCGN in different subpopulations of malignant cells.

Through the analysis of KIRC_GSE171306, we performed dimensionality reduction on the data (Figure 8A). MMP9 was highest expressed in monocytes/macrophages (Figures 8B, D). SCGN was highest expressed in malignant cells (Figures 8C, E). Because our subsequent functional experiments were conducted on SCGN, we selected SCGN for further single-cell transcriptome studies. We further classified the malignant cells where SCGN was located (Supplementary Figure S5A) and found that SCGN was highly abundant in subgroup 3 (Supplementary Figure S5B). We performed molecular function enrichment analysis, and the results showed that subgroup 3 was related to the transmembrane transport of multiple substances (Figure 8F). We conducted an enrichment analysis and subgroup 3 had the highest enrichment of metabolism-related pathways (Figure 8G). We performed pseudotime analysis in repartitioned cells and mapped the cell differentiation trajectories (Supplementary Figure S5C). Unfortunately, the expression of SCGN in malignant cells at different stages of differentiation did not change (Supplementary Figure S5D).

### SCGN increased the proliferation and invasion ability of tumor cells

**FIGURE 9:**
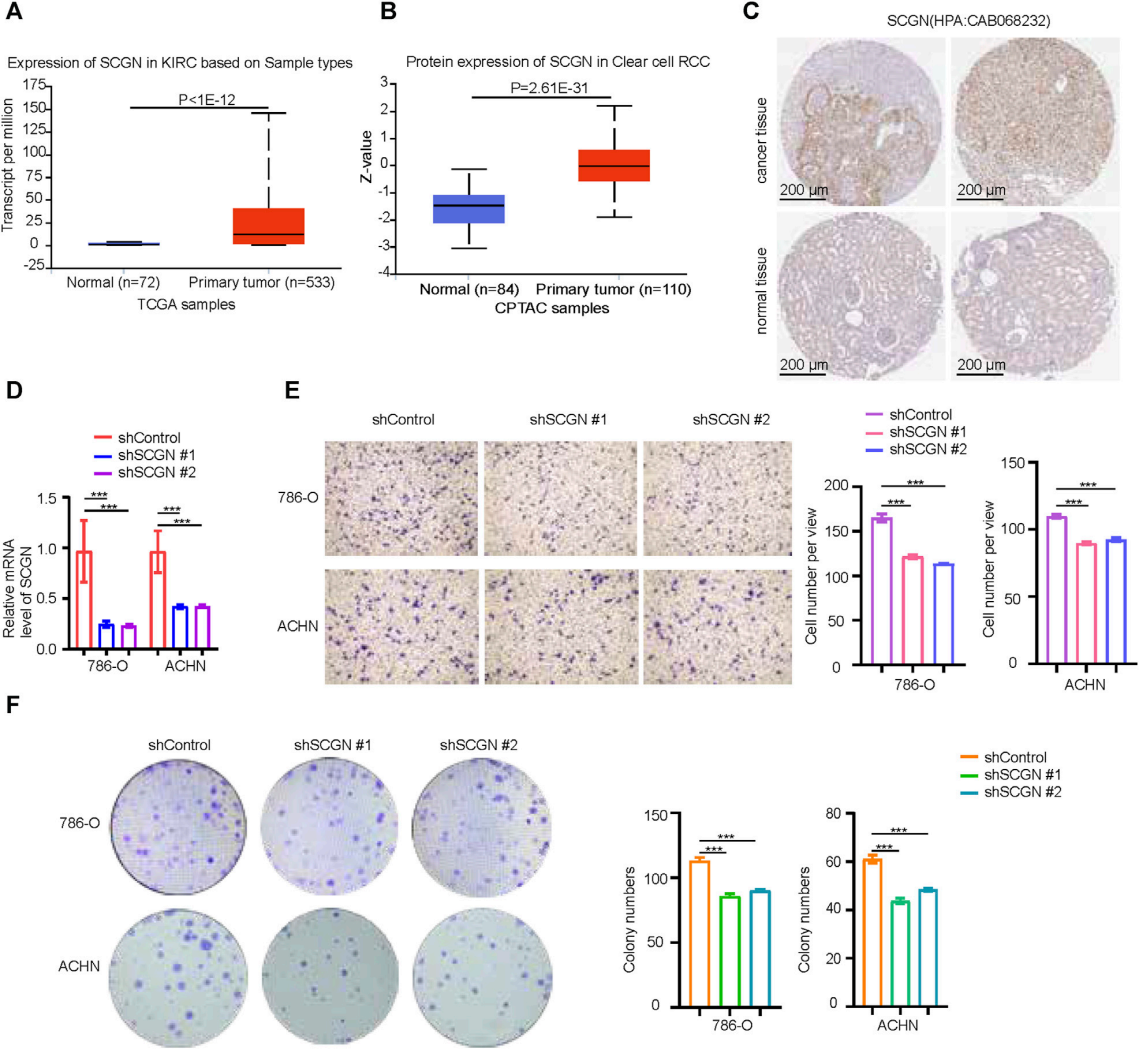
SCGN increases the proliferation and invasion ability of tumor cells. **(A, B)** SCGN protein and mRNA expression levels in normal and tumor tissues, from UALCAN (https://ualcan.path.uab.edu/). **(C)** Comparison of IHC staining of SCGN in tumor tissue and normal tissue, from HPA (https://www.proteinatlas.org/), Anti-body:CAB068232. **(D)** After knocking down SCGN in 786-O and ACHN cell lines, the relative expression of SCGN decreased. **(E)** Cell invasion was attenuated after knockdown of SCGN in 786-O and ACHN cell lines. **(F)** Knockdown of SCGN in 786-O and ACHN cell lines weakened cell proliferation. IHC, Immunohistochemistry.

The role of MMP9 in ccRCC had been thoroughly studied. Therefore, we performed validation on SCGN. At both the mRNA and protein levels, the expression of SCGN in tumor tissues was higher than that in normal tissues (Figures 9A, B), which was confirmed by IHC staining of HPA (Figure 9C). We performed knockdown of SCGN and verified the effect by RT-qPCR (Figure 9D). In addition, transwell experiments showed that the invasion ability of tumor cells was weakened after SCGN knockdown (Figure 9E). Through colony formation assays, we observed that tumor cell proliferation was weakened after SCGN knockdown (Figure 9F).

### Diagnostic genes were associated with prognosis of patients in ccRCC and with immunity in pan-cancer

We further explored the expression levels and prognosis of diagnostic molecules in ccRCC. MMP9 was upregulated in ccRCC tissues compared with normal tissues (Supplementary Figure S6A). Patients with high expression levels of MMP9 in ccRCC tissues had worse prognosis (Supplementary Figure S6B–F). SCGN expression was downregulated in ccRCC tissues compared with normal tissues (Supplementary Figure S6G). Patients with high expression of SCGN in ccRCC tissues had better prognosis (Supplementary Figure S6H–K). MMP9 and SCGN not only played regulatory roles in ccRCC, they had also shown value in pan-cancer. Compared with normal tissues, the expression levels of MMP9 and SCGN generally changed in pan-cancer (Supplementary Figure S7A). MMP9 and SCGN were generally associated with OS in pan-cancer, including ccRCC (Supplementary Figure S7B). In pan-cancer, both MMP9 (Supplementary Figure S8A) and SCGN (Supplementary Figure S9A) were associated with a variety of immune regulatory molecules, including chemokines, receptors, MHC molecules, immunosuppressive molecules, and immune activating molecules. In pan-cancer, both MMP9 (Supplementary Figure S10A) and SCGN (Supplementary Figure S10B) were associated with multiple immune checkpoint molecules. MMP9 and SCGN were poorly associated with microsatellite instability (MSI) in pan-cancer (Supplementary Figures S10C, D). MMP9 and SCGN were associated with a variety of immune cells (Supplementary Figures S11A, B).

## Discussion

The advent of immunotherapy has undoubtedly enhanced the prognosis of ccRCC patients. However, a significant proportion of patients remain unresponsive to immunotherapy, warranting the identification of patients suitable for immunotherapy (Luo et al., 2019).

Collagen, being a major protein component of the ECM, plays a multifaceted role in both intracellularly and extracellularly (Phang et al., 2008; Wu et al., 2021). Previous studies have shown that clear cell renal cell carcinoma can be divided into different subtypes from different perspectives (Bai et al., 2021; Yang et al., 2023). However, the contribution of collagen to ccRCC classification remains unknown. Our study distinguishes two distinct ccRCC subtypes, named subtype 1 and subtype 2.

Results of enrichment analysis show that the differences between the two subtypes are mainly in immunity and tumorigenesis, so we continue the analysis. Subtype 2 exhibits higher infiltration of immune cells and stromal components compared to subtype 1. High levels of exhausted immune cell infiltration are associated with poorer prognosis in ccRCC patients (Peng et al., 2020; Braun et al., 2021). Prior studies have indicated elevated cytotoxic T lymphocyte (CTL) levels and an enrichment of T cell dysfunction in ccRCC, leading to enhanced tumor immune evasion through a more severe degree of T cell dysfunction (Jiang et al., 2018). It has also been shown that in ccRCC, CXCL13+CD8^+^ T cell abundance impairs total CD8^+^ T cell function, and CXCL13+CD8^+^ T cell infiltration indicates poorer clinical outcomes in ccRCC patients (Dai et al., 2021). Our further analysis reveals that subtype 2 exhibited higher TIDE score and expressions of molecules related to immune evasion and T cell exhaustion. Consequently, we hypothesize that despite subtype 2’s higher immune cell infiltration, immune escape may prevail due to immune cell dysfunction and overexpression of immune checkpoint. In the immunotherapy cohort, even though immune cells in subtype 2 are widely infiltrated, the prognosis of subtype 2 is poor. This also illustrates the stability of immune cell infiltration in different subtypes. Regardless of the TMB level (high or low), the prognosis of subtype 1 is superior to that of subtype 2. The new subtypes we identified can be a good addition to patient selection.

Based on subtype 1 and subtype 2, using core genes to build a diagnostic model makes it easier to determine the patients’ subtype. The discrimination and calibration of our model are relatively good. Although the analysis of immune-related indicators of the two subtypes indicates that subtype 2 is prone to immune escape, clinical evidence is lacking. Clinical cohort validation shows that subtype 2 patients receiving anti-PD-L1 therapy have shorter PFS. We believe that in the absence of differences in tumor mutational burden, tumor heterogeneity between the two subtypes partially contributes to differences in the immune system’s ability to kill tumor cells.

MMP-9 is upregulated in ccRCC (Ma et al., 2020). In ccRCC, an increasing number of studies have shown that MMP9 promotes tumor invasion and migration (Wu et al., 2019; Wang J. et al., 2020; Zhang et al., 2022). High expression level of MMP9 is associated with poor prognosis in patients with ccRCC (Niu et al., 2018). MMP9 affect the survival of circulating tumor cells in clear cell renal cell carcinoma by adapting to tumor immune microenvironment (Guo et al., 2023). Besides, a study shows that in ccRCC, MMP9 can regulate tumor immunity (Xu et al., 2021). The inhibition of MMP2/MMP9 by SB-3CT prolongs survival time by promoting anti-tumor immunity (Ye et al., 2020). Yiming Lu et al. find the MMP9+ macrophages to be terminally differentiated tumor-associated macrophages (TAMs) (Lu et al., 2022). Our single-cell transcriptome analysis also showed that MMP9 is mainly expressed in monocytes/macrophages. Secretagogin (SCGN), a calcium-sensor protein, promotes the expression of matrix metalloprotease 2 (MMP2) in neurons (Qin et al., 2020). Loss of SCGN can lead to activation of inflammation (Liu et al., 2023). In the context of cancer, SCGN has emerged as a novel marker for cervical neuroendocrine carcinoma and has been linked to sorafenib resistance in hepatocellular carcinoma (Yu et al., 2021; Wang et al., 2022). SCGN protein is detected in kidney cancer samples but not in normal tissues (Kim et al., 2010). One study shows that SCGN is associated with tumor metastasis in ccRCC (Ilhan et al., 2011). Recent study has shown that SCGN has the potential to become an indicator for ccRCC subtype classification (Lai et al., 2023). Metabolites such as amino acids secreted by tumor cells can affect the status of immune cells in the microenvironment, but the impact is complex (Mellman et al., 2023). Our results indicate that SCGN may affect tumor response to immunotherapy by regulating various metabolisms of tumor cells.

The pan-cancer analysis we performed also shows that the diagnostic molecules are associated with many immune molecules and immune cells, but not with MSI. Both in ccRCC and other tumors, the diagnostic molecules are associated with immune-activating and immunosuppressive molecules or cells, suggesting that they may be involved in complex immune regulation within the tumor.

## Conclusion

In summary, we construct two new molecular subtypes of ccRCC and a diagnostic model based on subtype-specific marker molecules to define the subtype to which patients belong. These may help doctors to select more suitable patients for immunotherapy.

## Data Availability

The datasets presented in this study can be found in online repositories. The names of the repository/repositories and accession number(s) can be found in the article/Supplementary Material.

## Abbreviations

AUC, Area Under the Curve; BPR, boxplot of Residuals; CNV, copy number variation; ccRCC, clear cell renal cell carcinoma; EGB, extreme gradient boosting; ECM, extracellular matrix; DEGs, differential expression genes; GO, Gene Ontology; GSEA, Gene set enrichment analysis; GLM, generalized linear model; KEGG, Kyoto Encyclopedia of Genes and Genomes; MSI, microsatellite instability; NMF, non-negative matrix factorization; OS, Overall Survival; PPI, Protein-Protein Interaction; PFS, progression free survival; RCDP, reverse cumulative distribution plot; ROC, receiver operating characteristic; RCC, renal cell carcinoma; SVM, support vector machine; ssGSEA, single-sample GSEA; RF, random forest; TMB, tumor mutation burden; TISCH, Tumor Immune Single-cell Hub; TIDE, tumor immunedysfunctionandexclusion; WGCNA, weighted gene co-expression network analysis; 33CRDs, 33 collagen-related differentially expressed genes.
