# Supplementary figures and images for: Identification of molecular subtypes and diagnostic model in clear cell renal cell carcinoma based on collagen-related genes may predict the response of immunotherapy
Source: Front Pharmacol. 2024 Feb 5;15:1325447. doi: 10.3389/fphar.2024.1325447 (PMC10875022; doi:10.3389/fphar.2024.1325447)

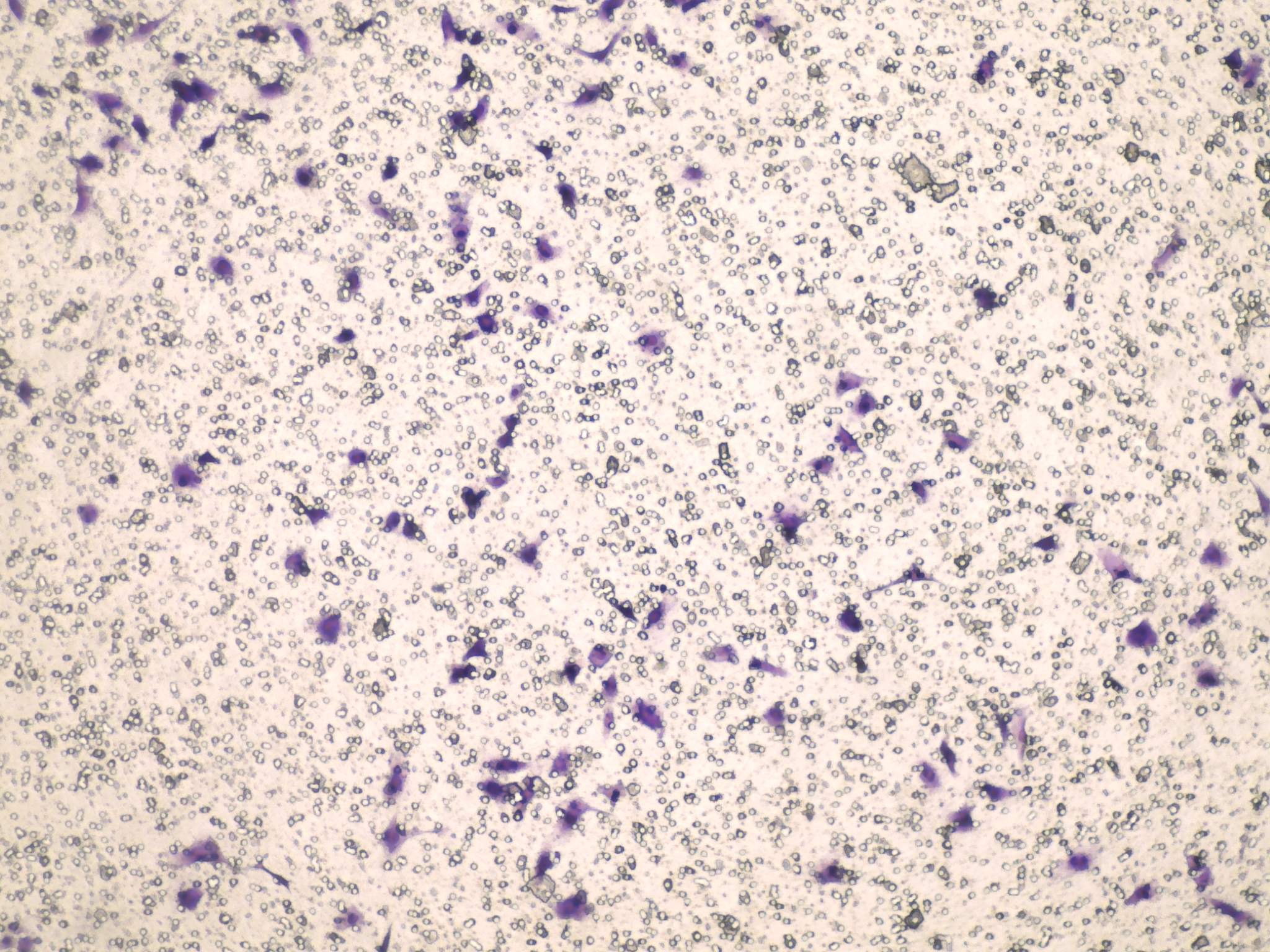

Supplement: Supplementary file 1 [file DataSheet3.zip › rawdata_transwell_786O/shSCGN1.tif]

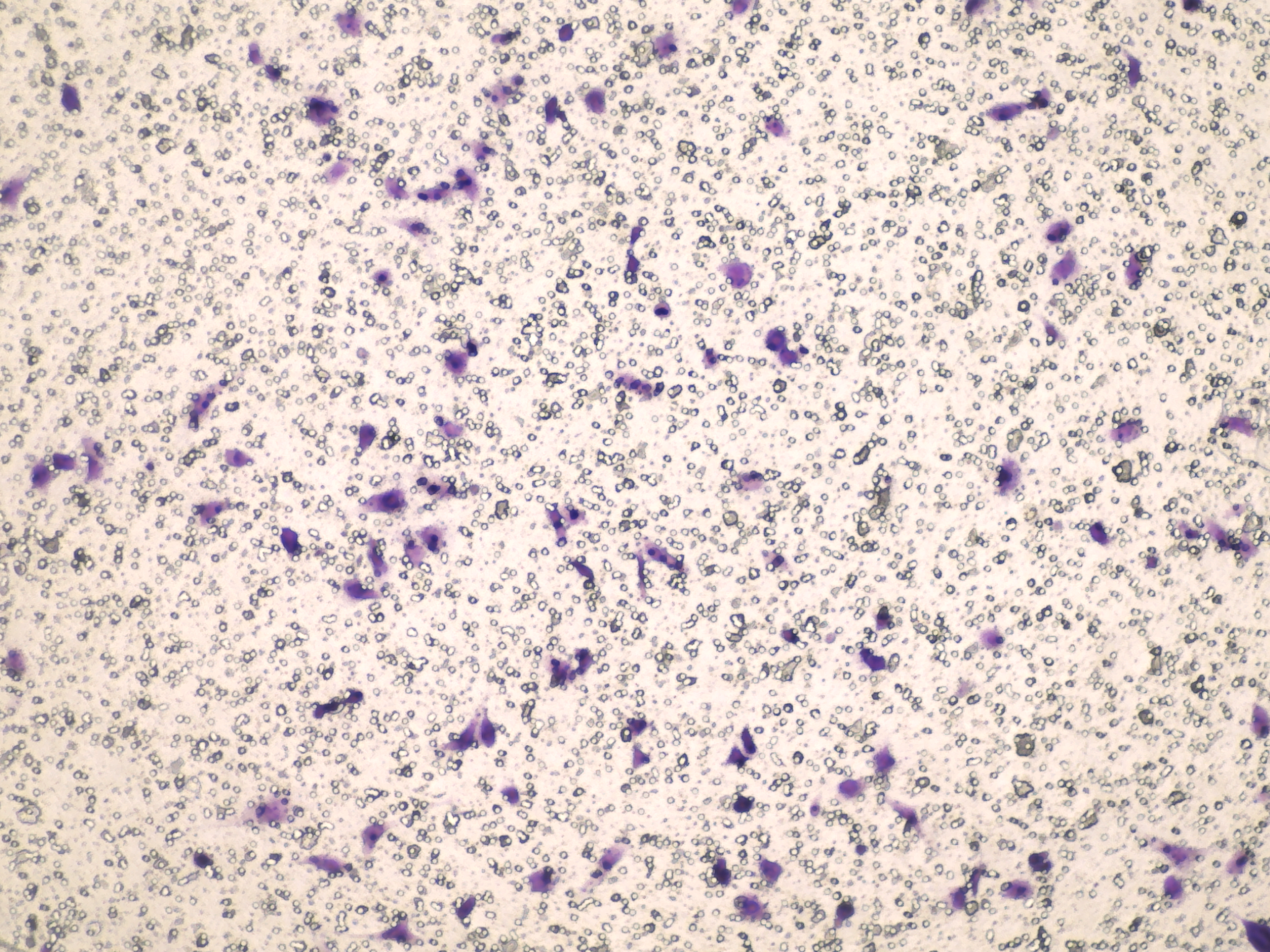

Supplement: Supplementary file 1 [file DataSheet3.zip › rawdata_transwell_786O/shSCGN2.tif]

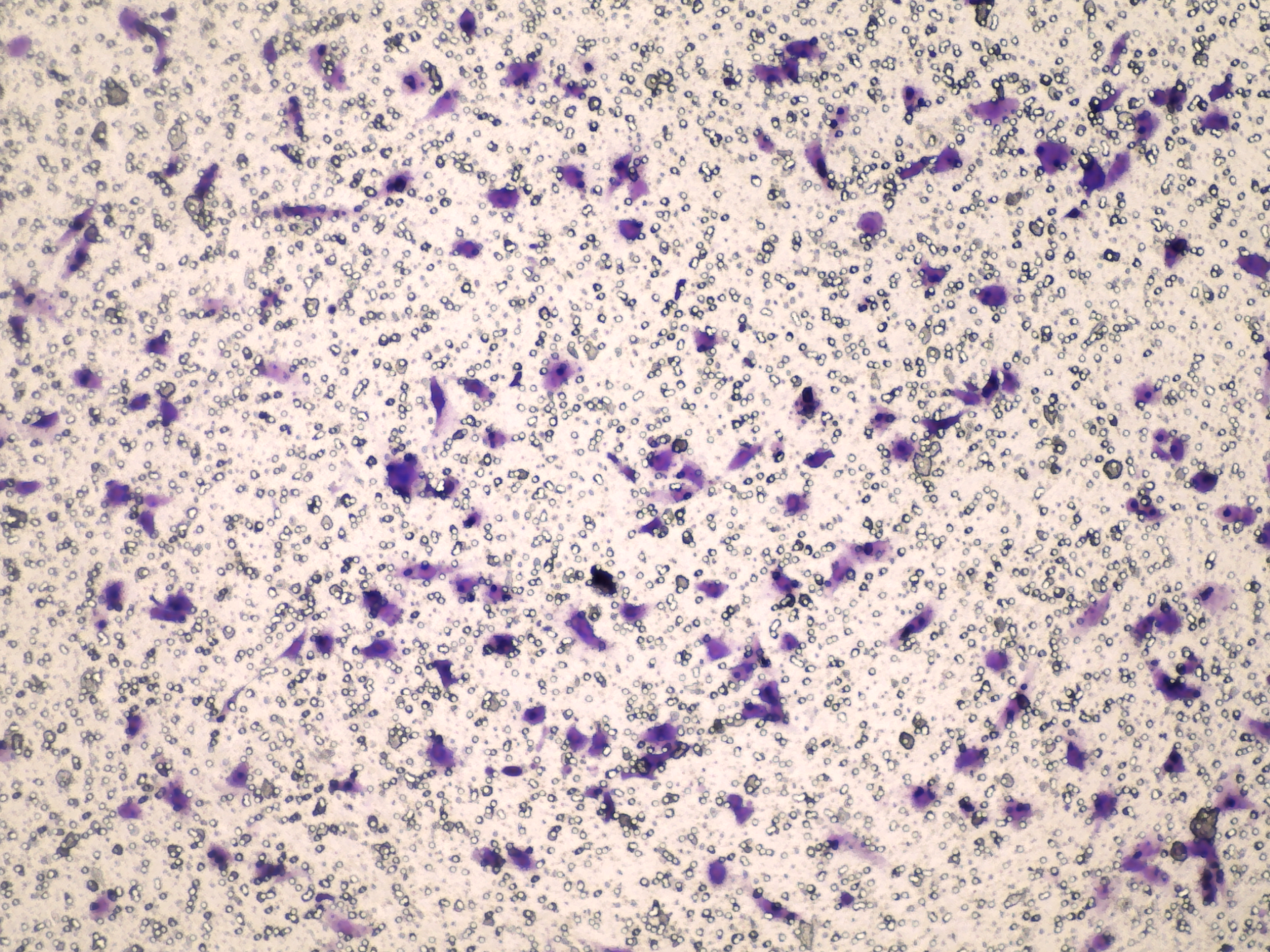

Supplement: Supplementary file 1 [file DataSheet3.zip › rawdata_transwell_786O/shSCGN╡─nc.tif]

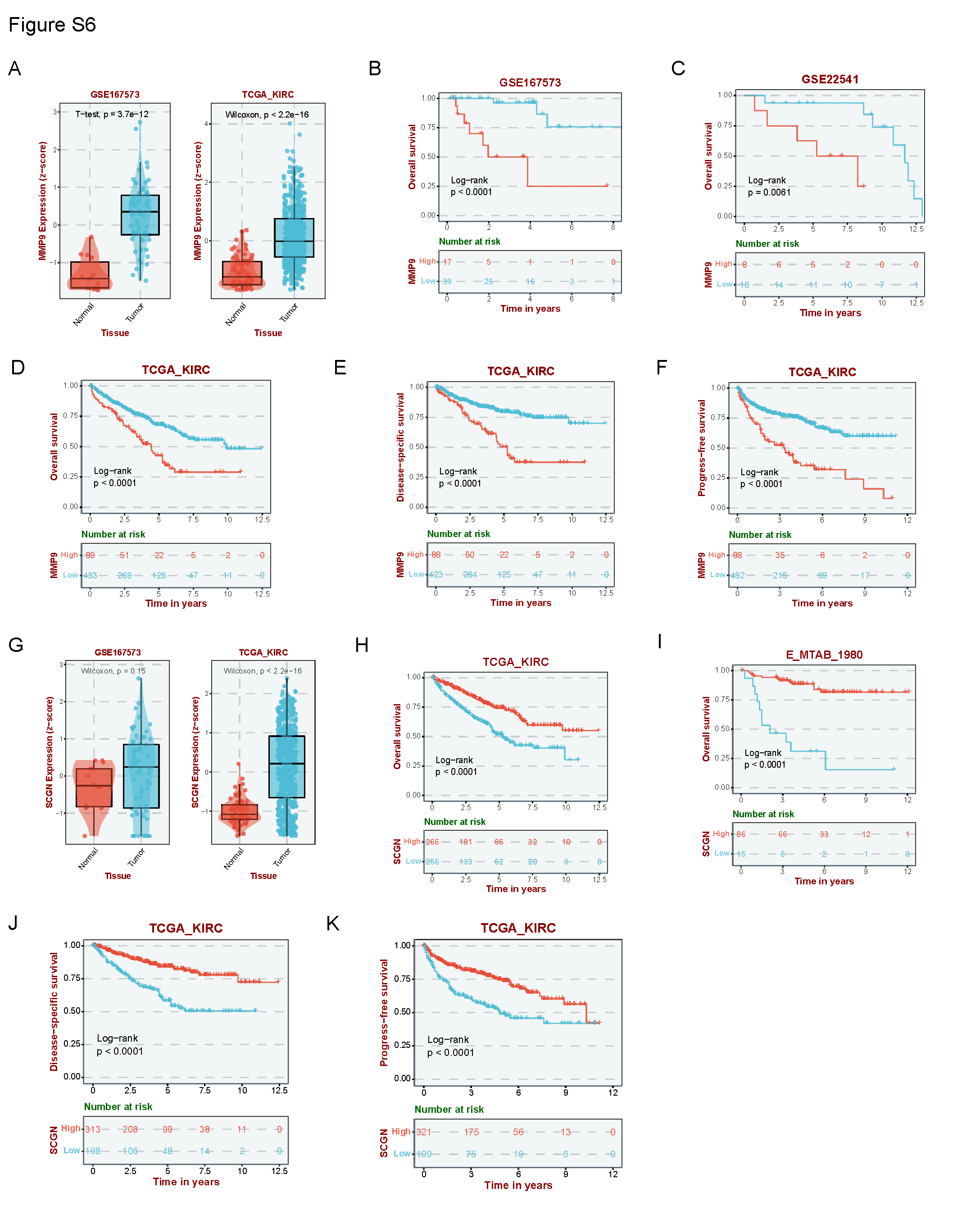

Supplement: Supplementary file 2 [file Image6.tif]

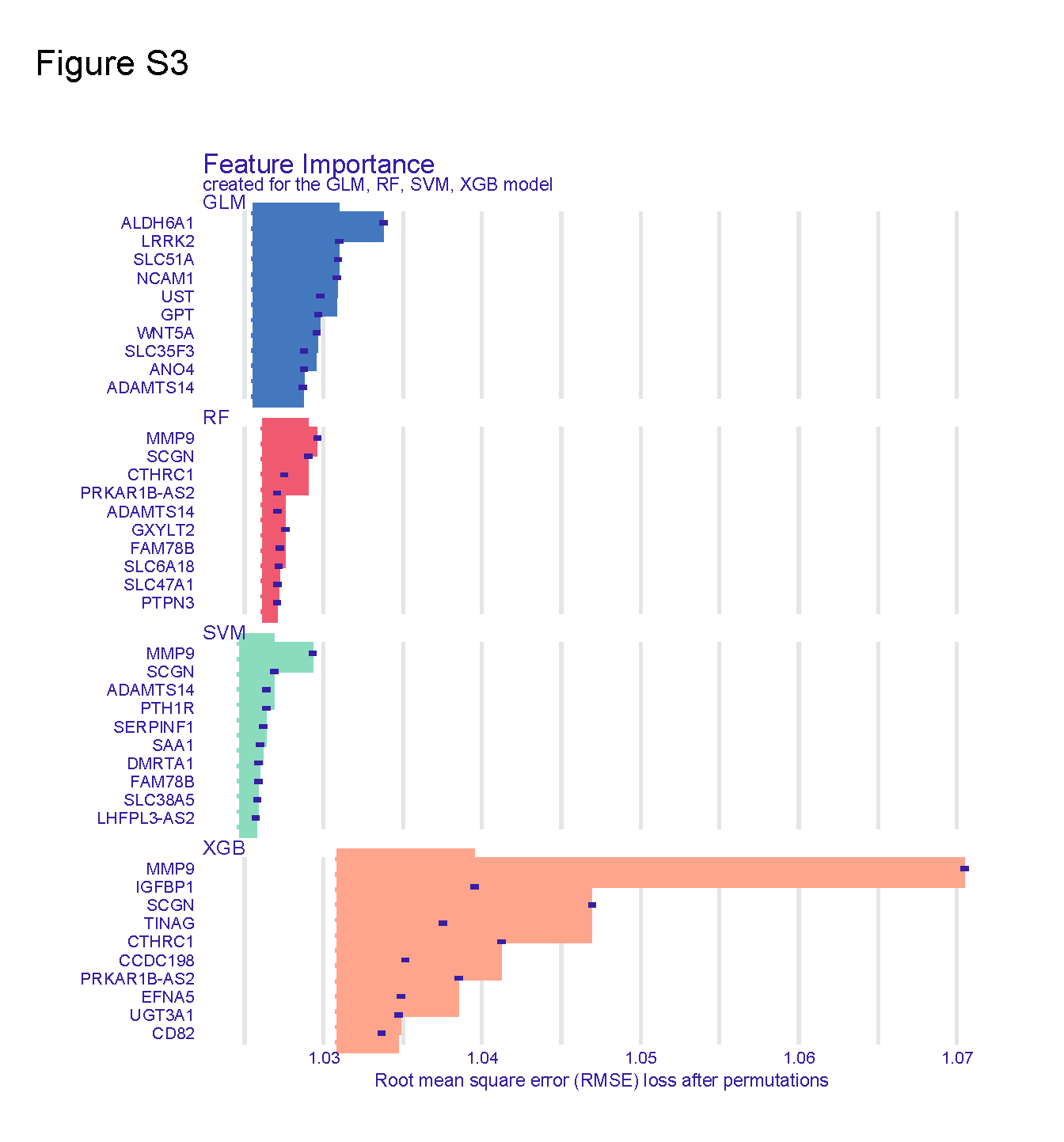

Supplement: Supplementary file 3 [file Image3.tif]

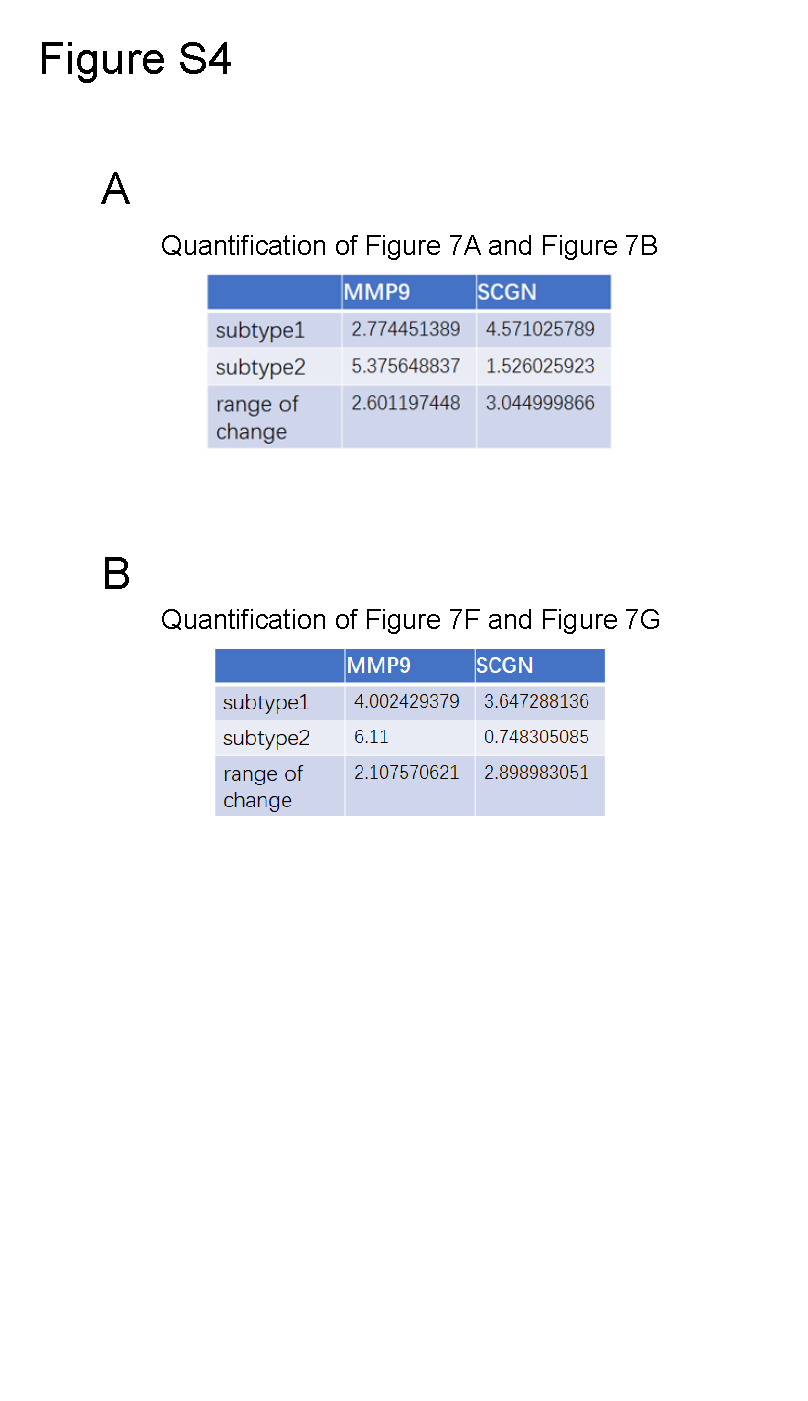

Supplement: Supplementary file 4 [file Image4.tif]

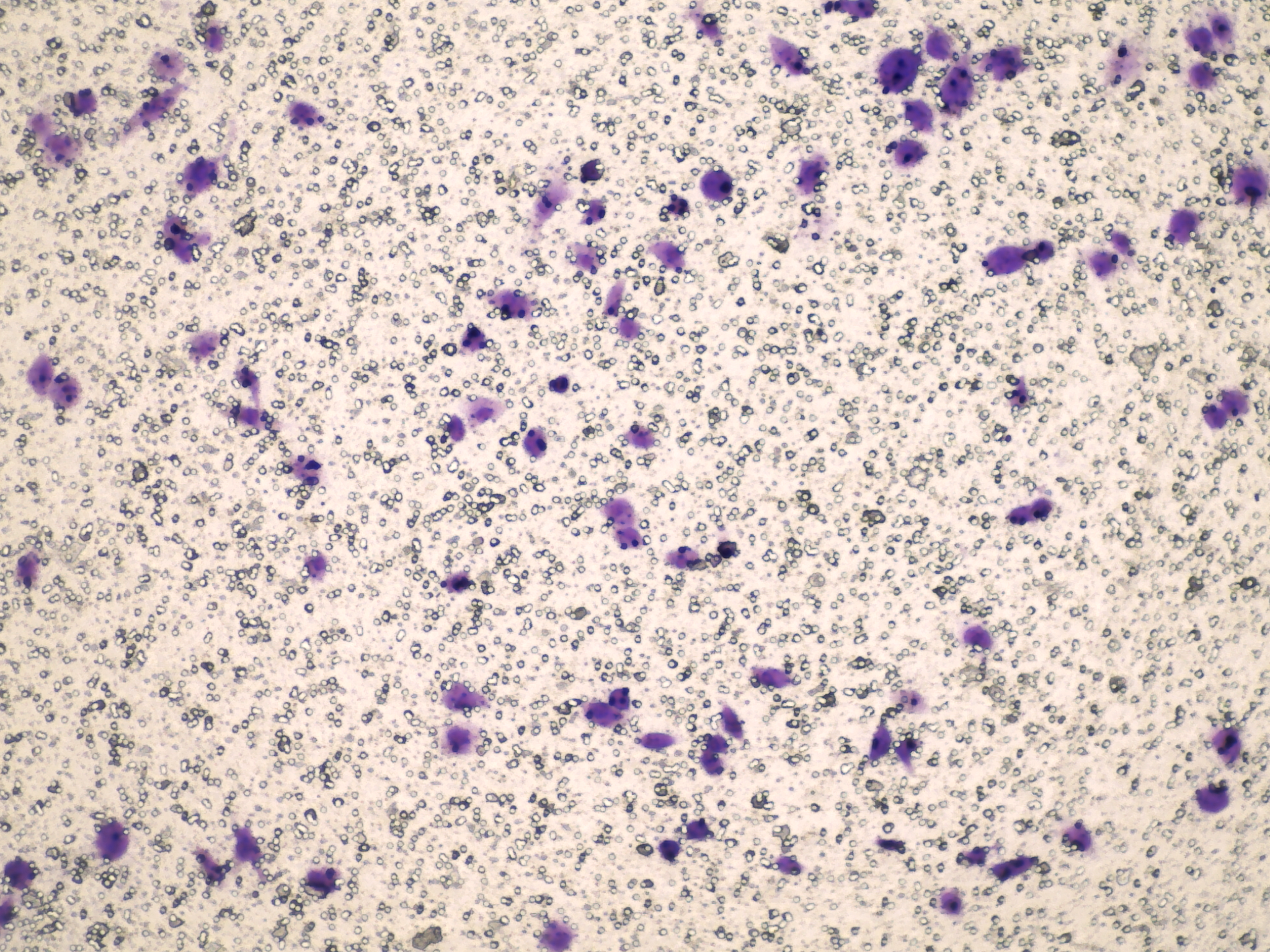

Supplement: Supplementary file 5 [file DataSheet4.zip › rawdata_transwell_ACHN/3w1.tif]

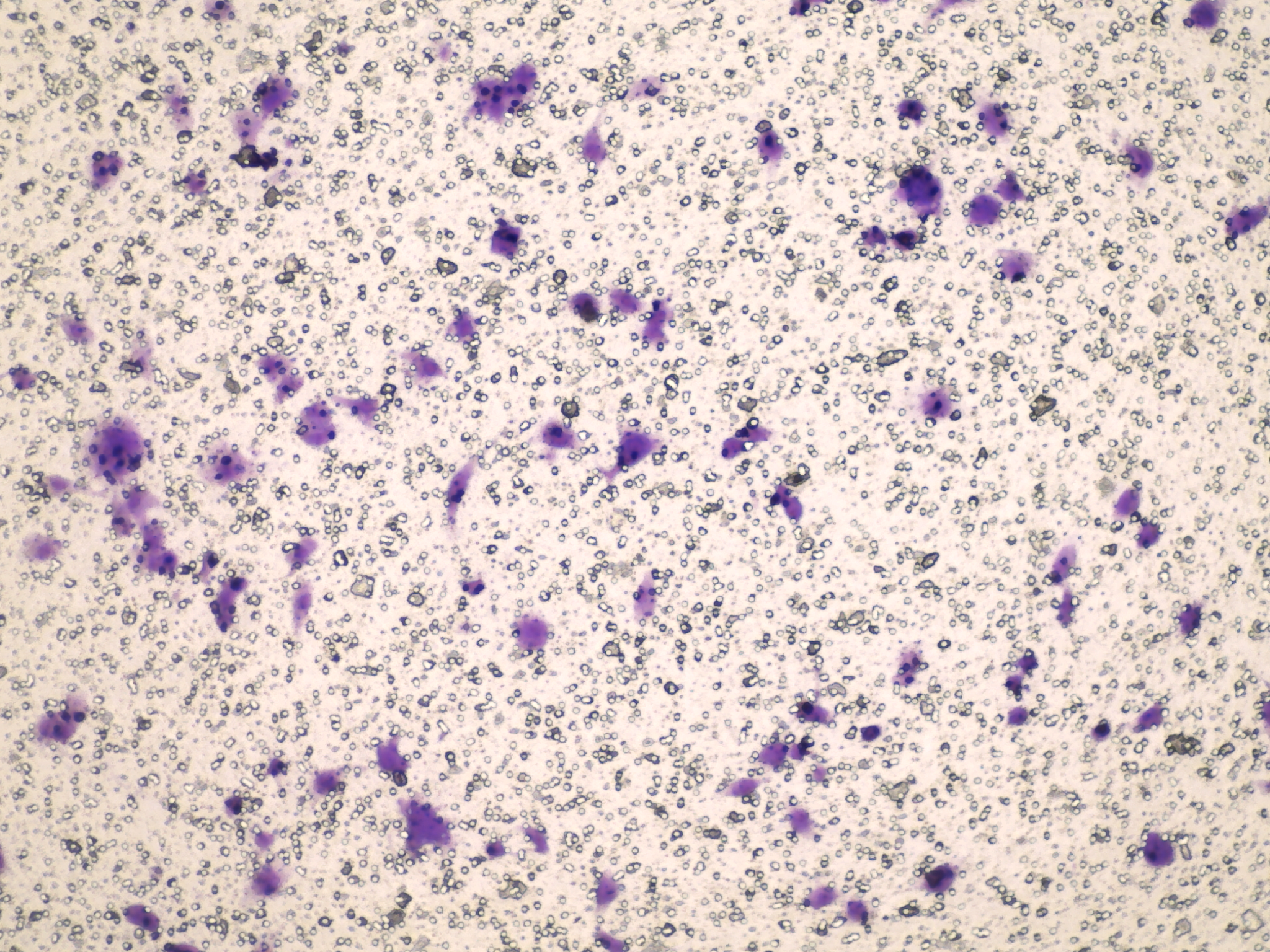

Supplement: Supplementary file 5 [file DataSheet4.zip › rawdata_transwell_ACHN/4w1.tif]

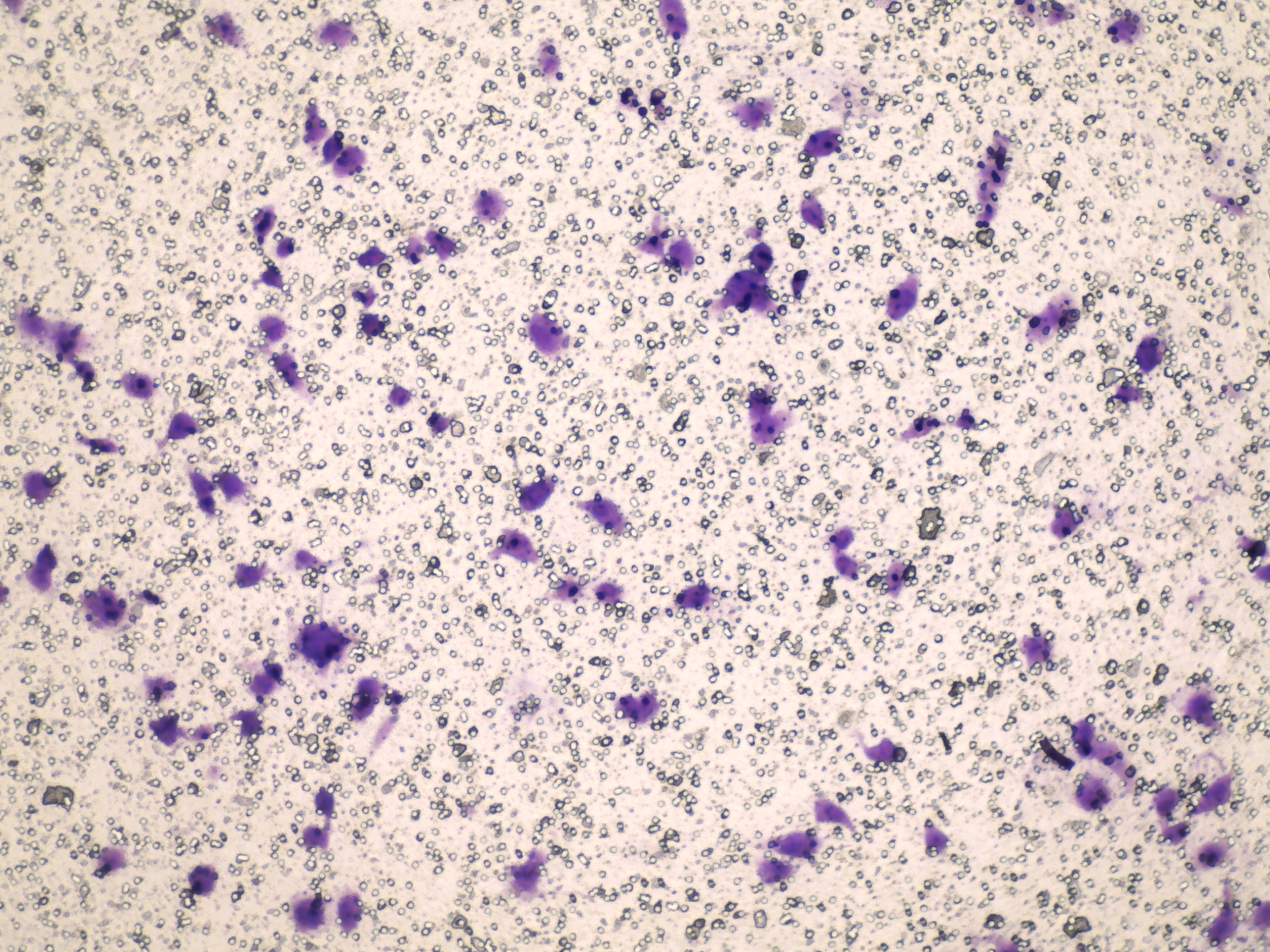

Supplement: Supplementary file 5 [file DataSheet4.zip › rawdata_transwell_ACHN/5w1.tif]

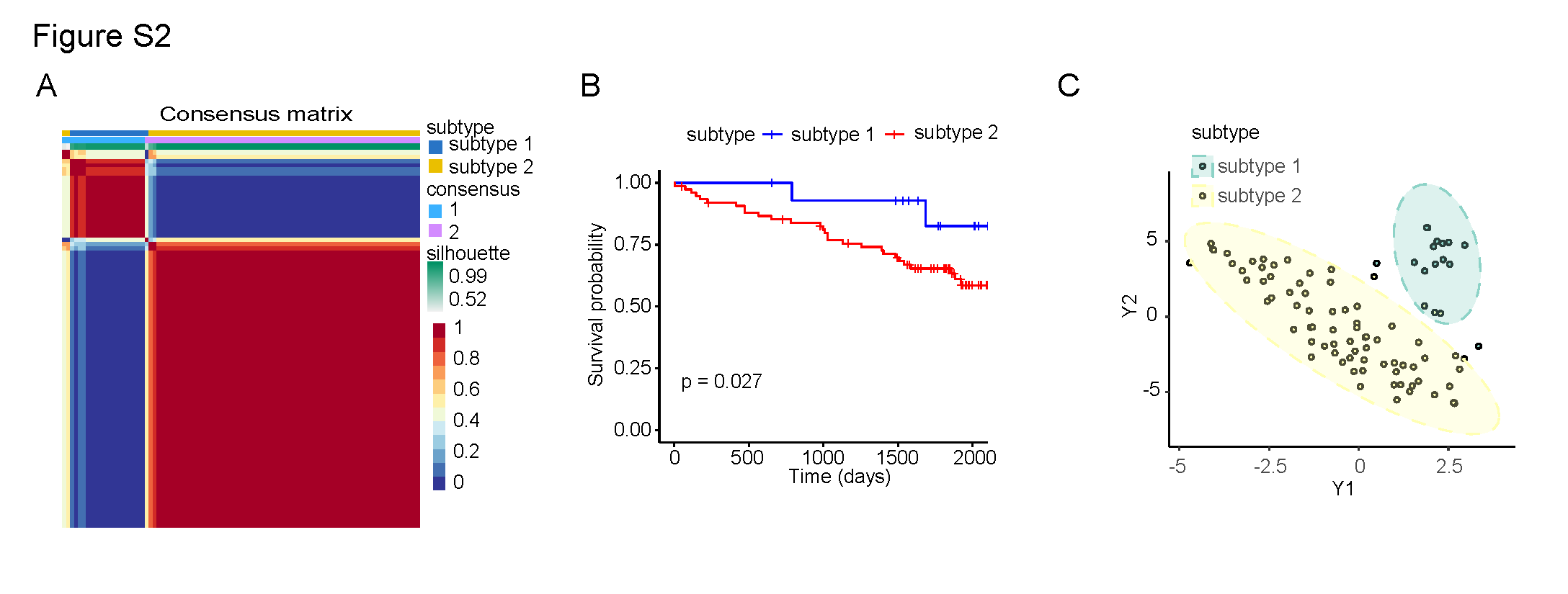

Supplement: Supplementary file 7 [file Image2.tif]

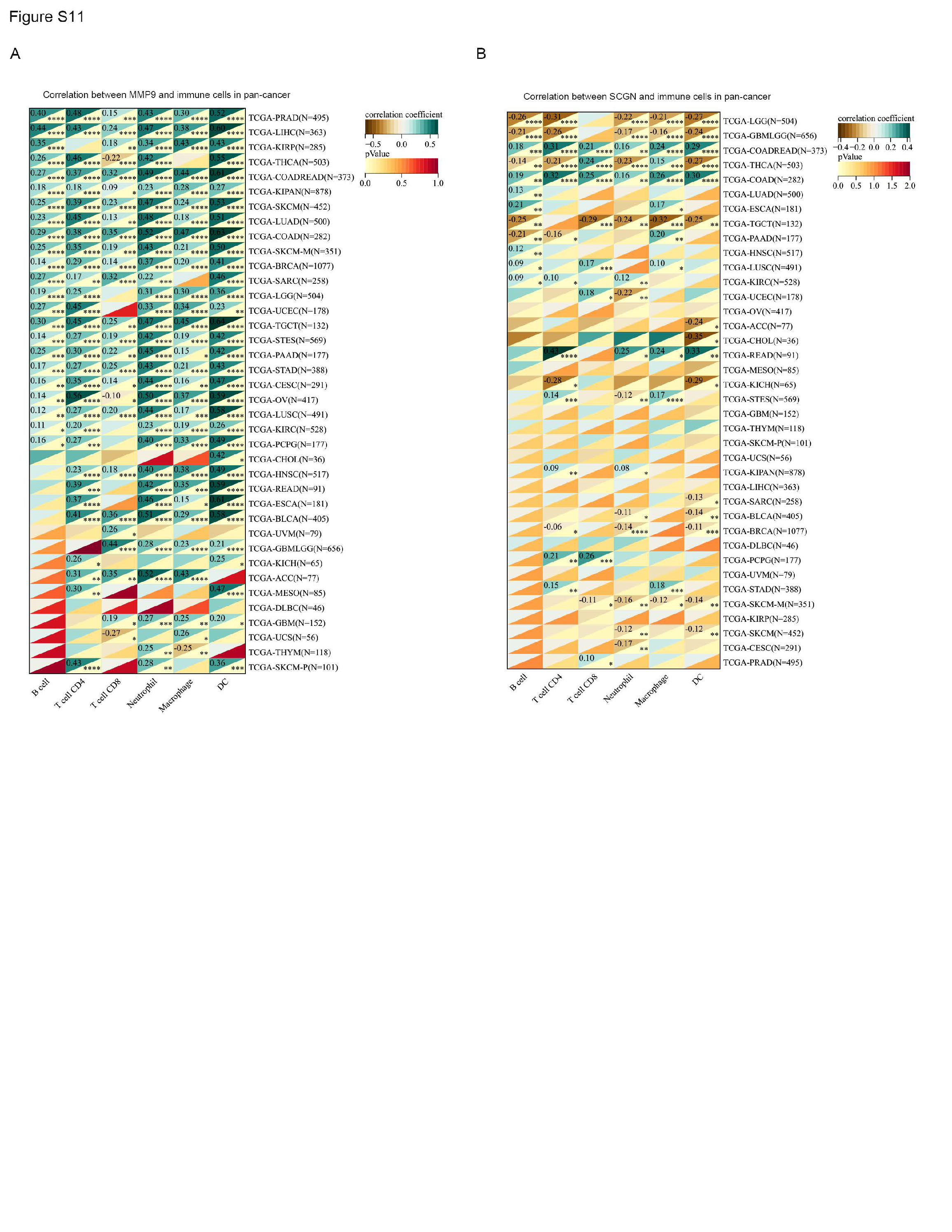

Supplement: Supplementary file 8 [file Image11.tif]

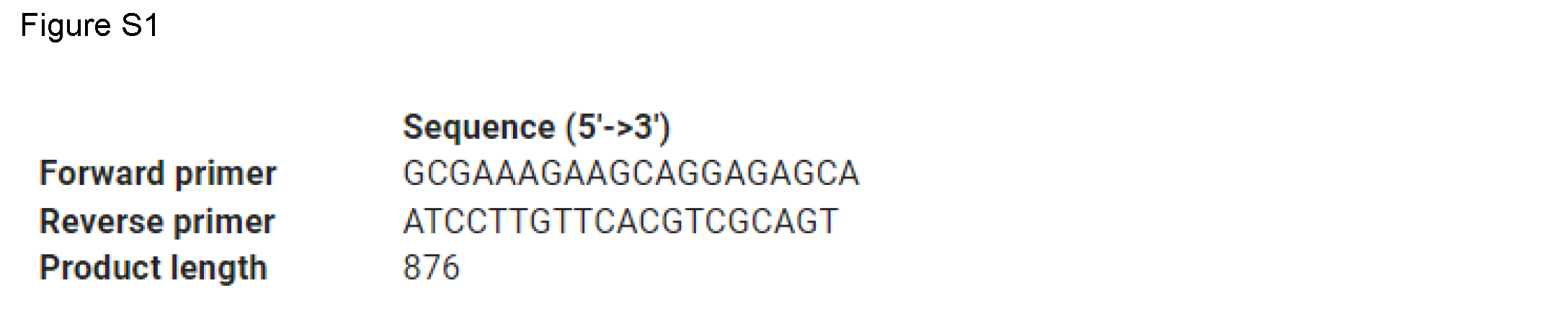

Supplement: Supplementary file 9 [file Image1.tif]

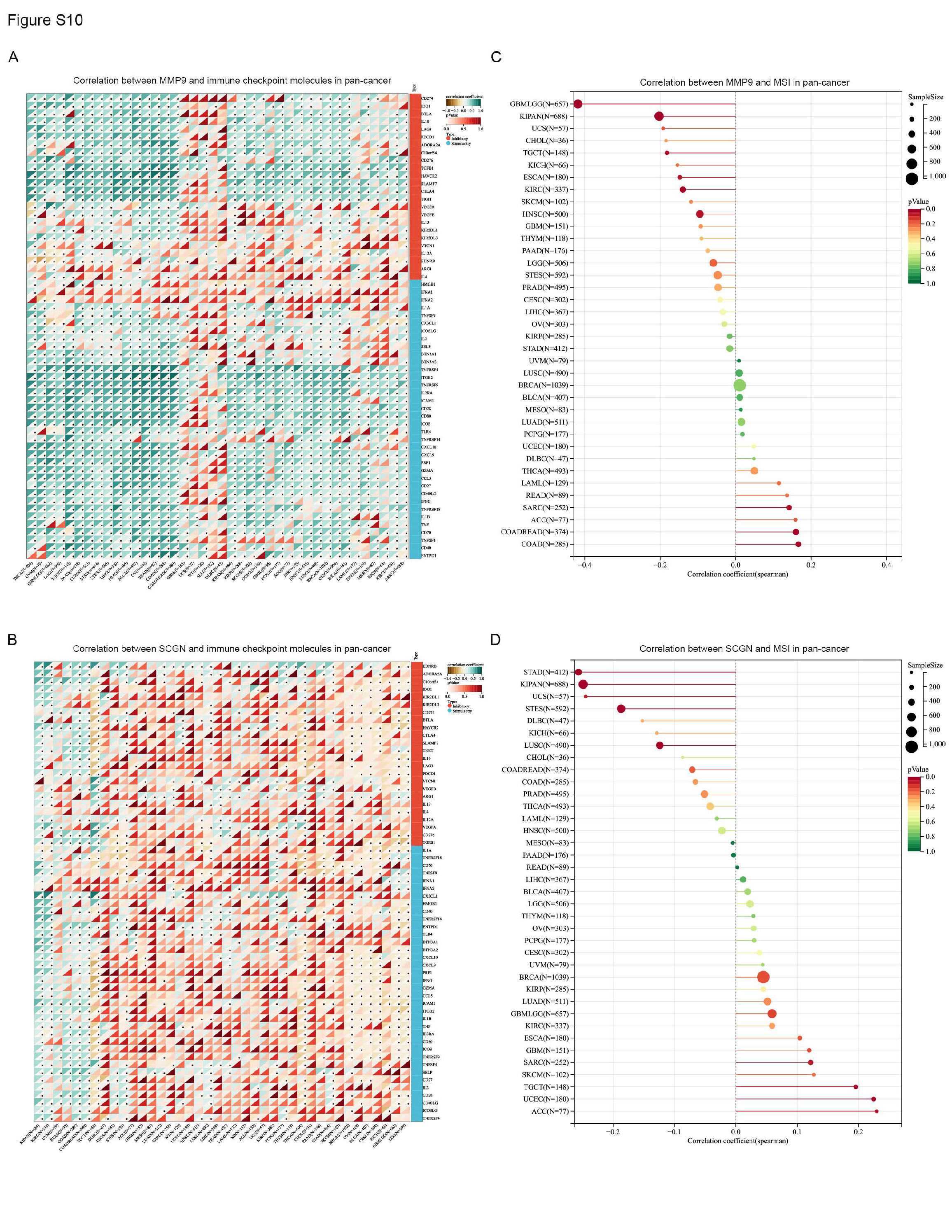

Supplement: Supplementary file 10 [file Image10.tif]

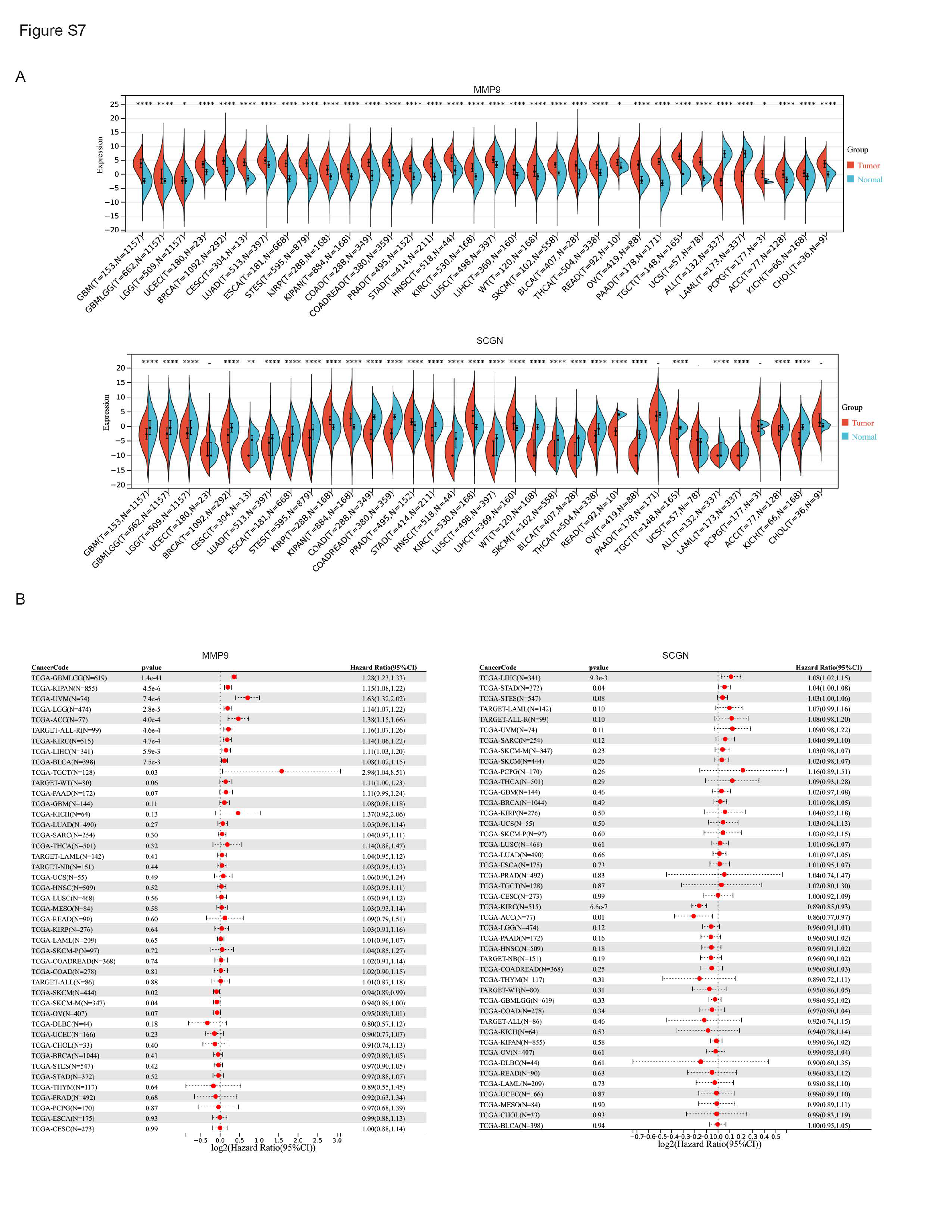

Supplement: Supplementary file 11 [file Image7.tif]

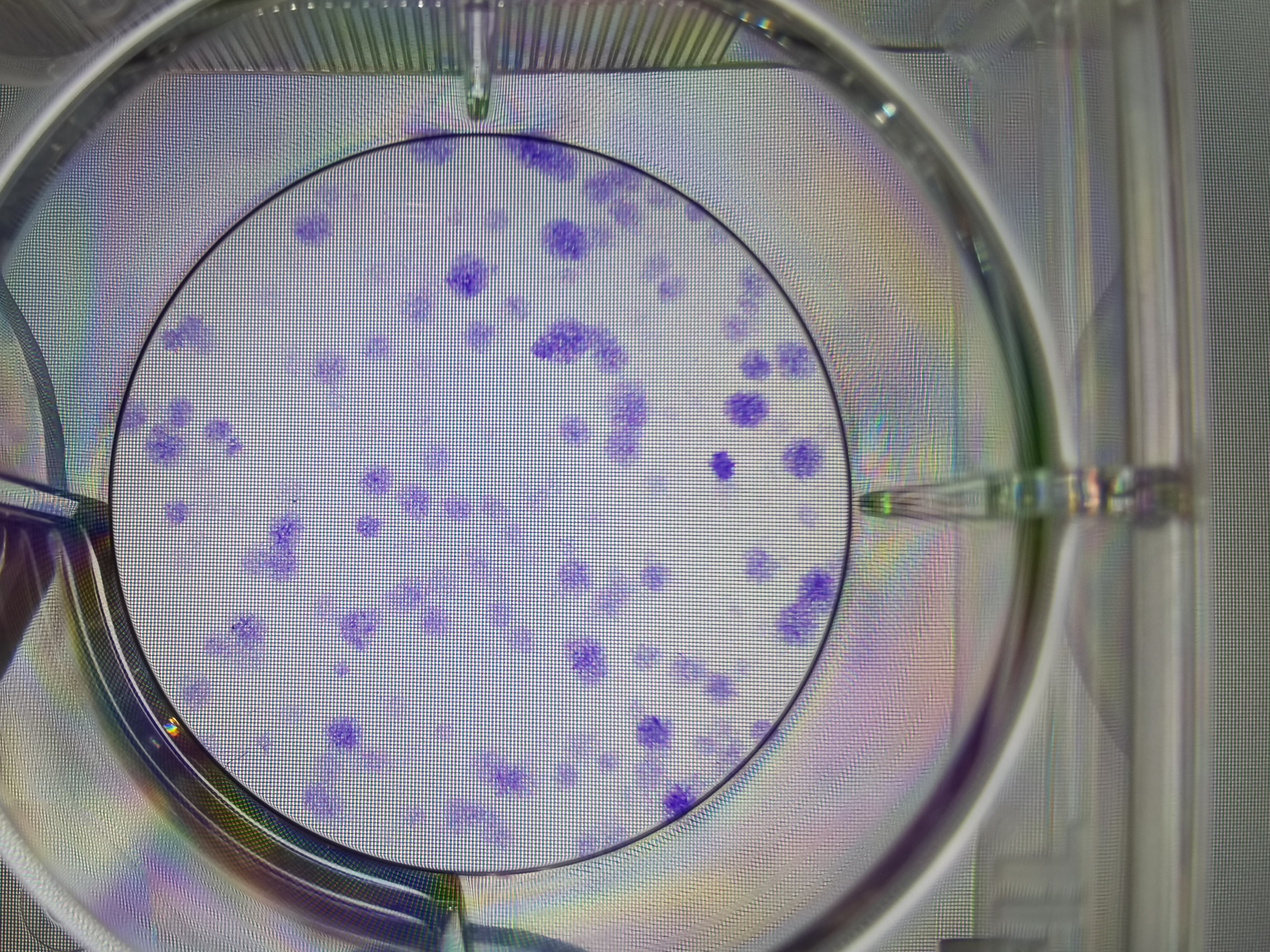

Supplement: Supplementary file 12 [file DataSheet2.zip › rawdata_clony formation/786OSCGN#2.jpg]

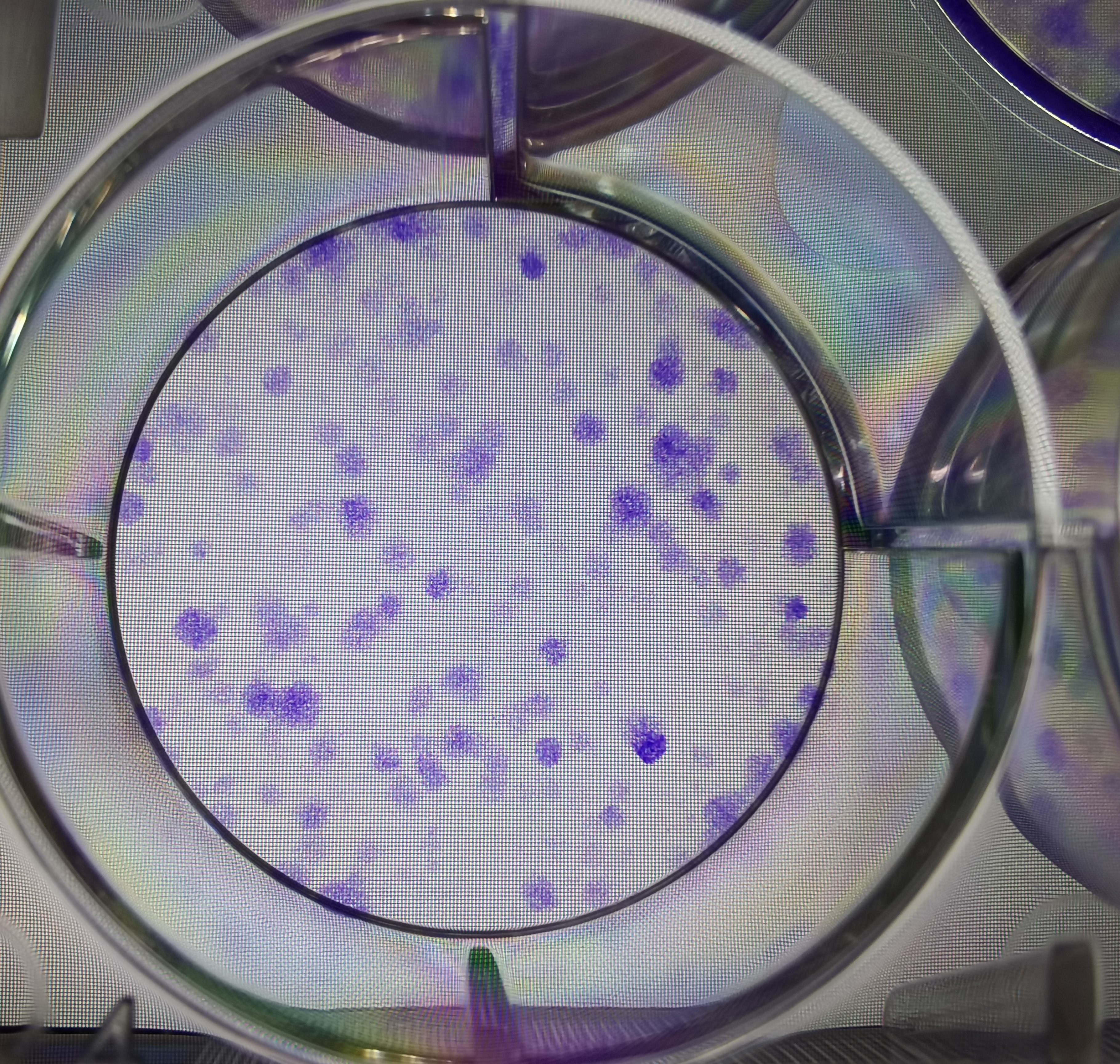

Supplement: Supplementary file 12 [file DataSheet2.zip › rawdata_clony formation/786Oshcontrol.jpg]

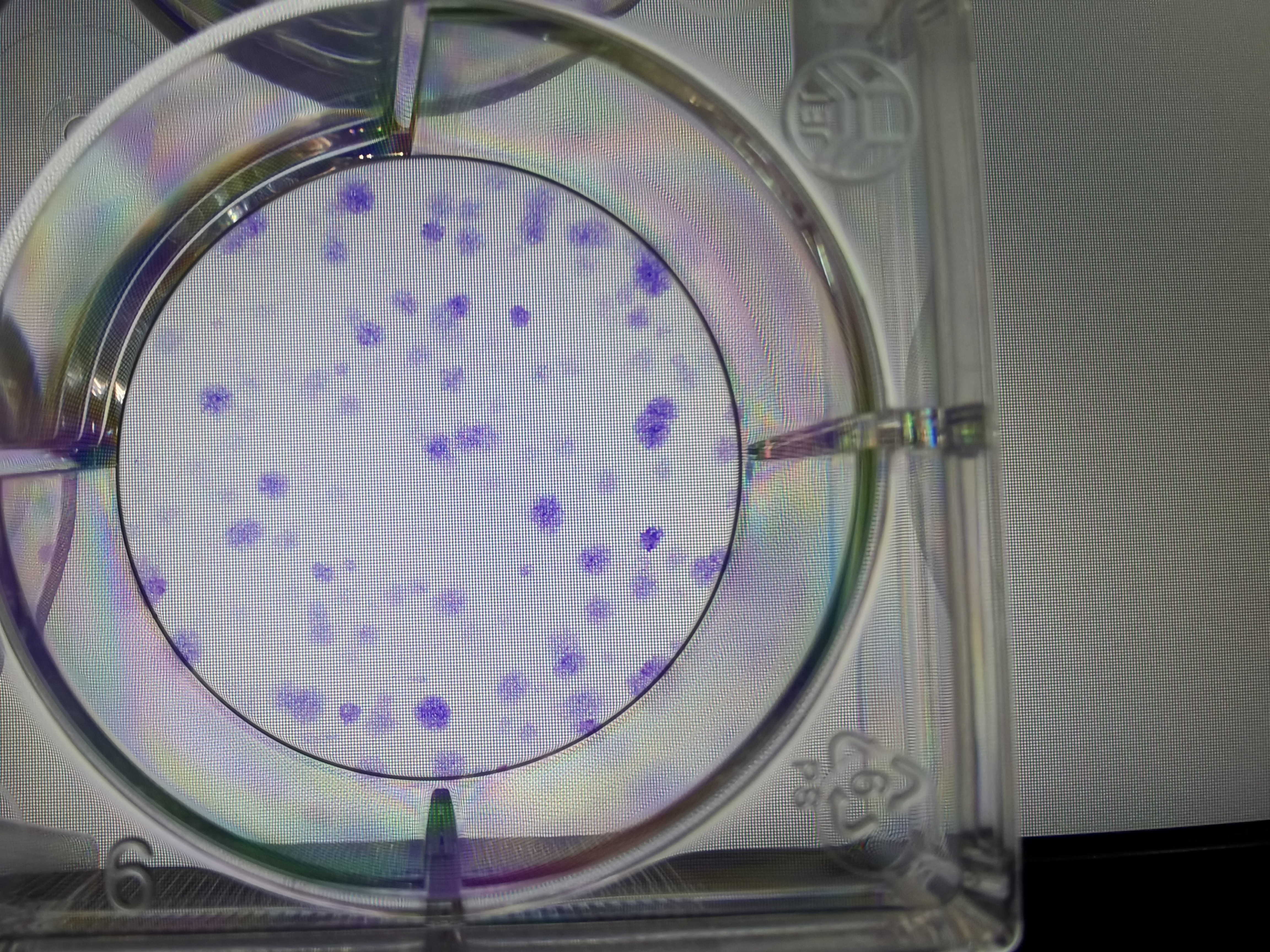

Supplement: Supplementary file 12 [file DataSheet2.zip › rawdata_clony formation/786OshSCGN#1.jpg]

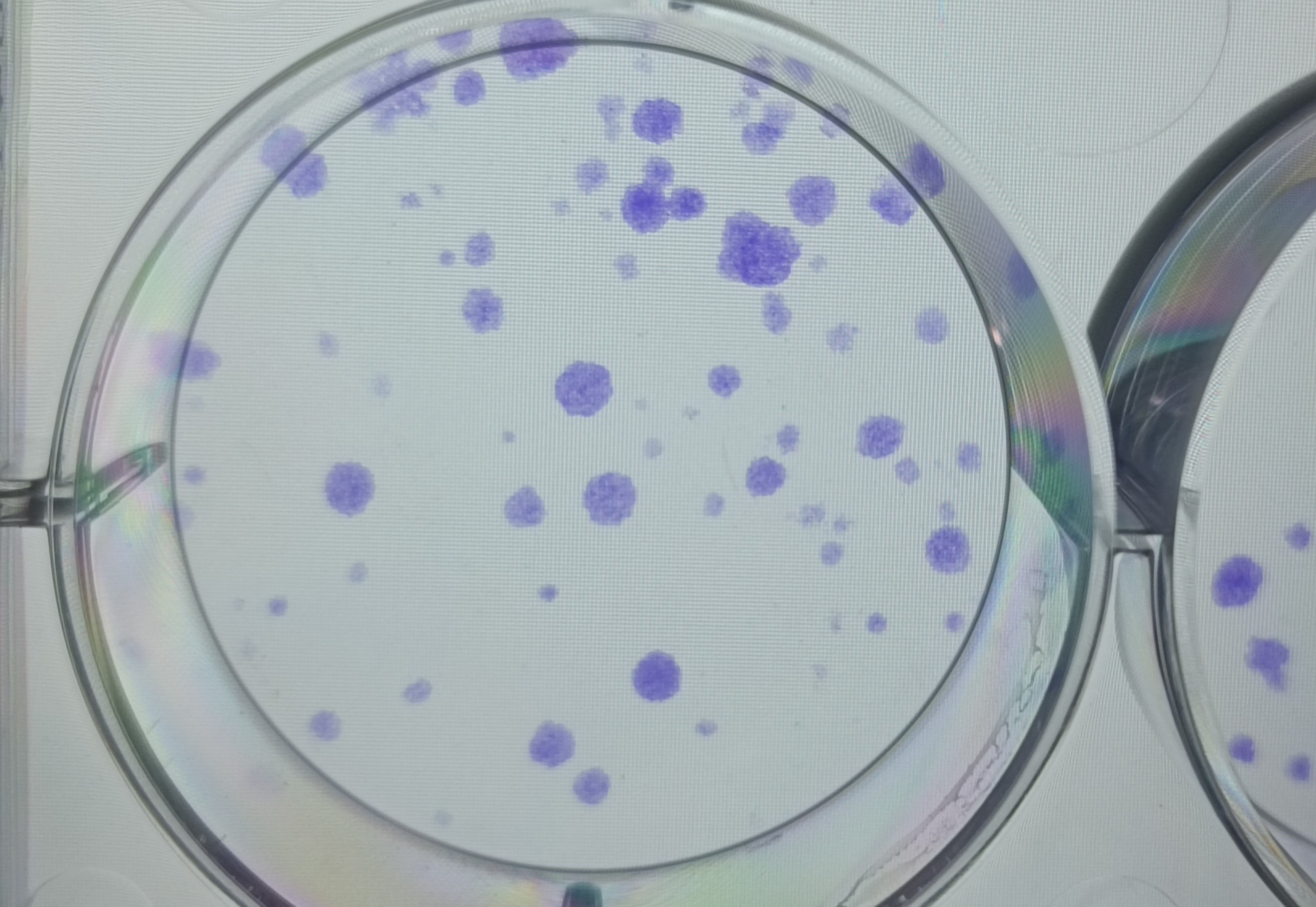

Supplement: Supplementary file 12 [file DataSheet2.zip › rawdata_clony formation/ACHNshcontrol.jpg]

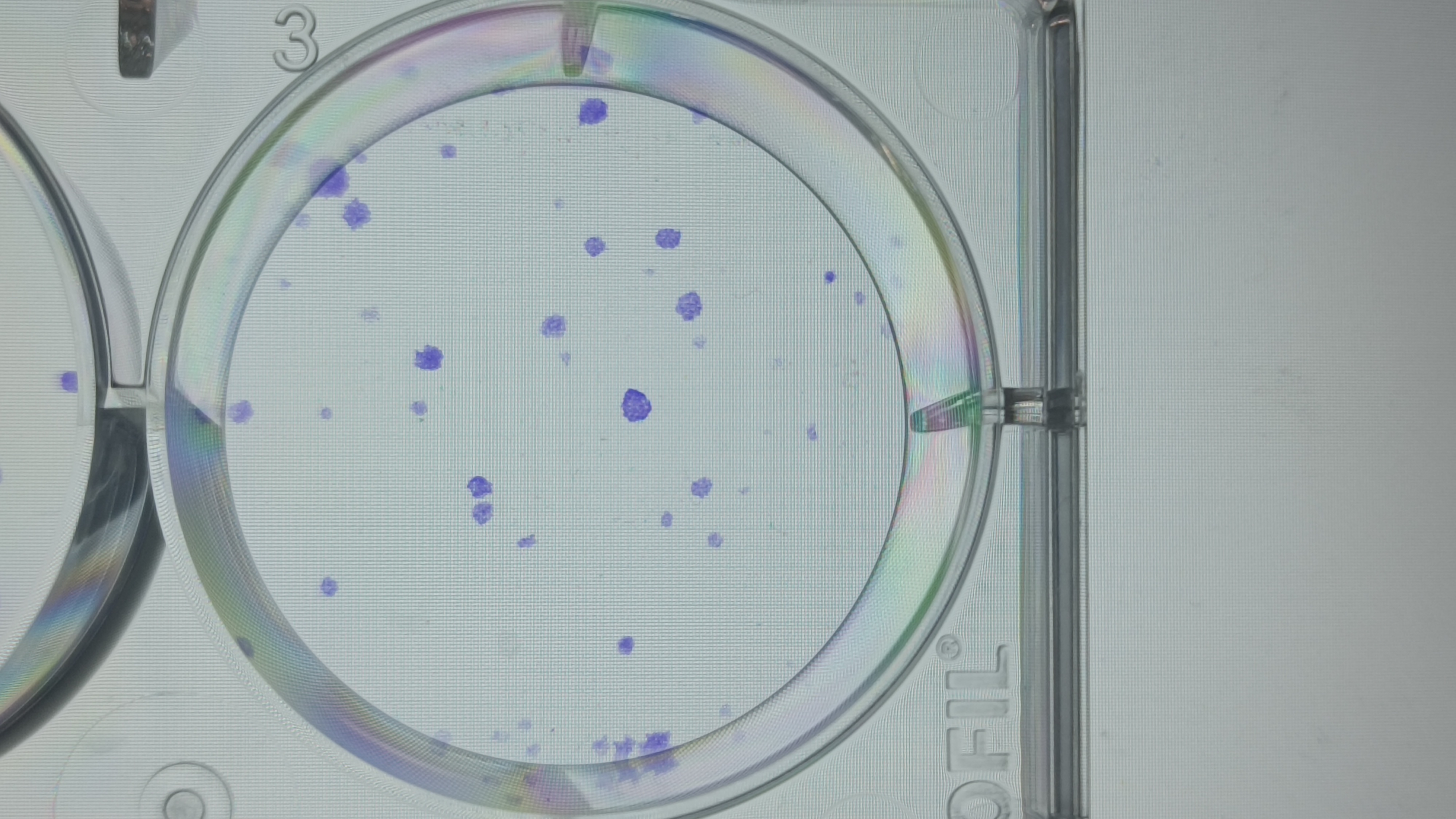

Supplement: Supplementary file 12 [file DataSheet2.zip › rawdata_clony formation/ACHNshSCGN#1.jpg]

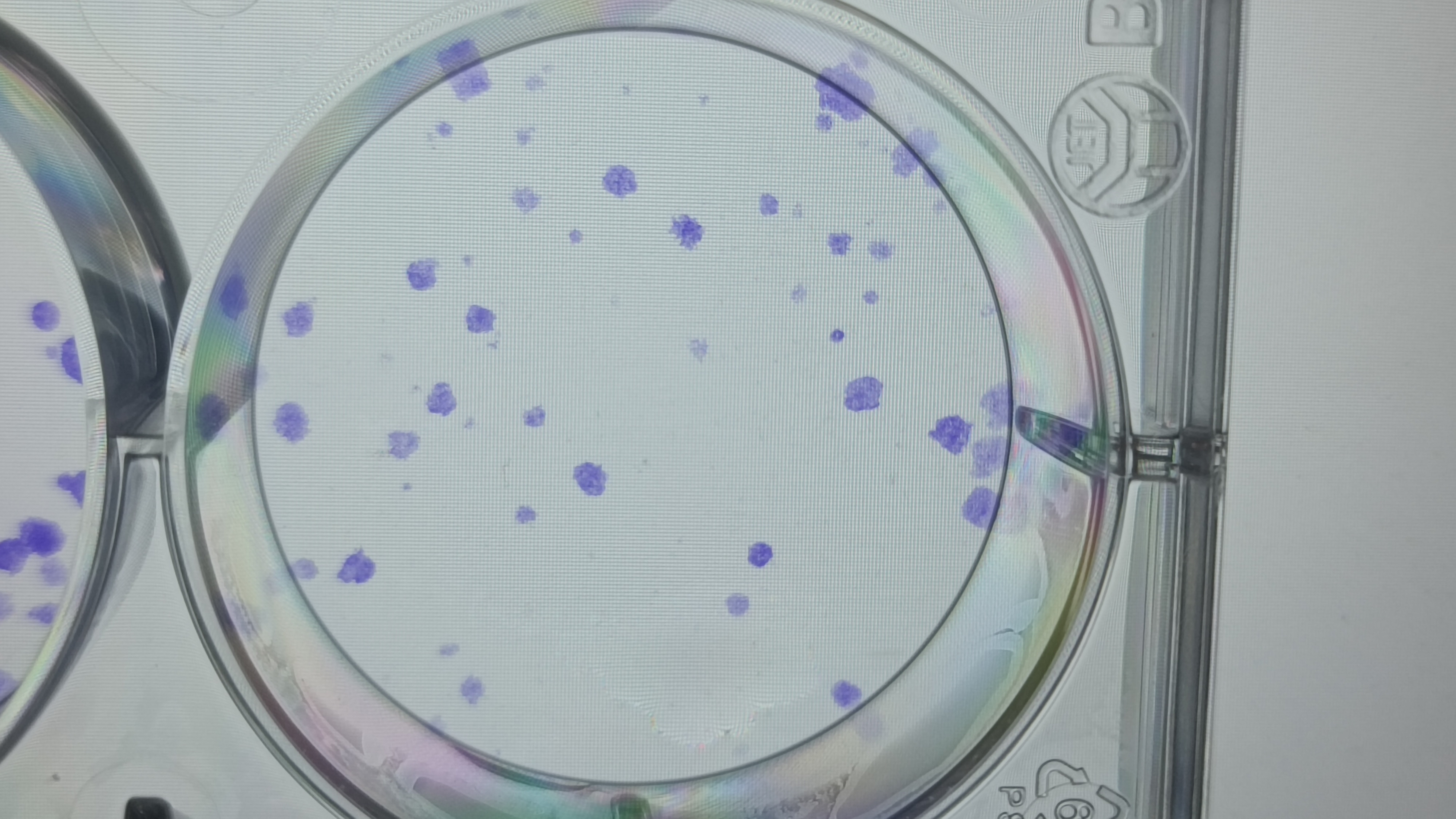

Supplement: Supplementary file 12 [file DataSheet2.zip › rawdata_clony formation/ACHNshSCGN#2.jpg]

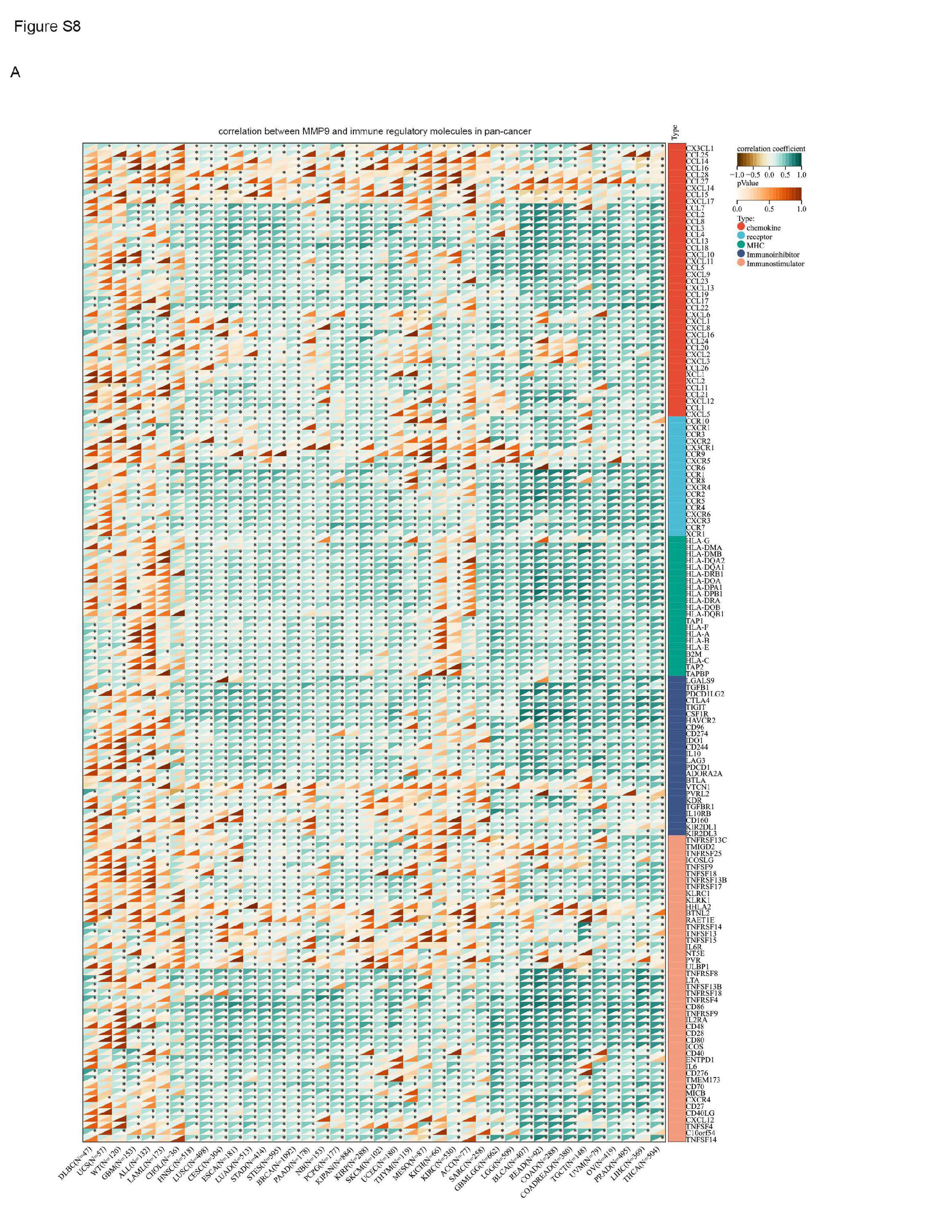

Supplement: Supplementary file 13 [file Image8.png]

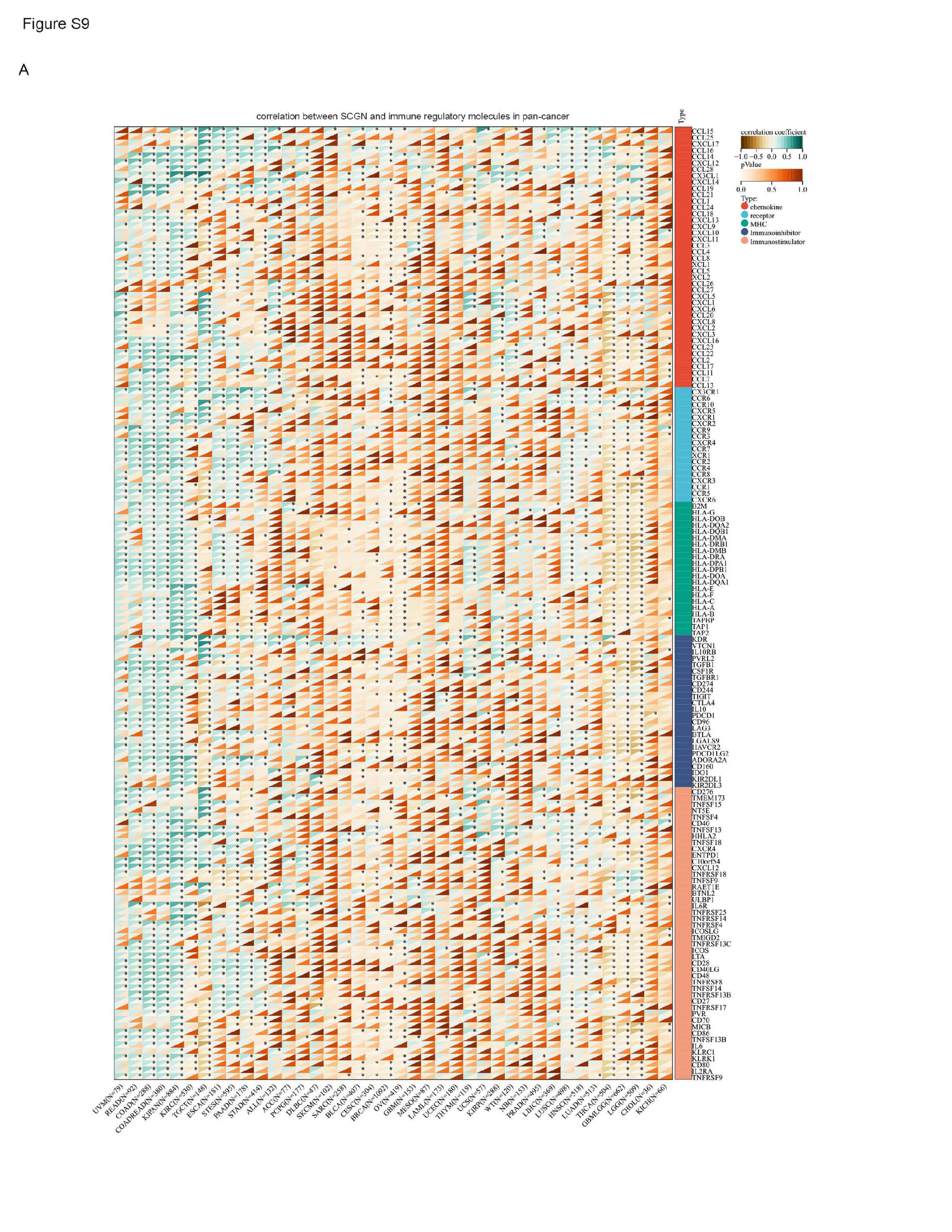

Supplement: Supplementary file 14 [file Image9.png]

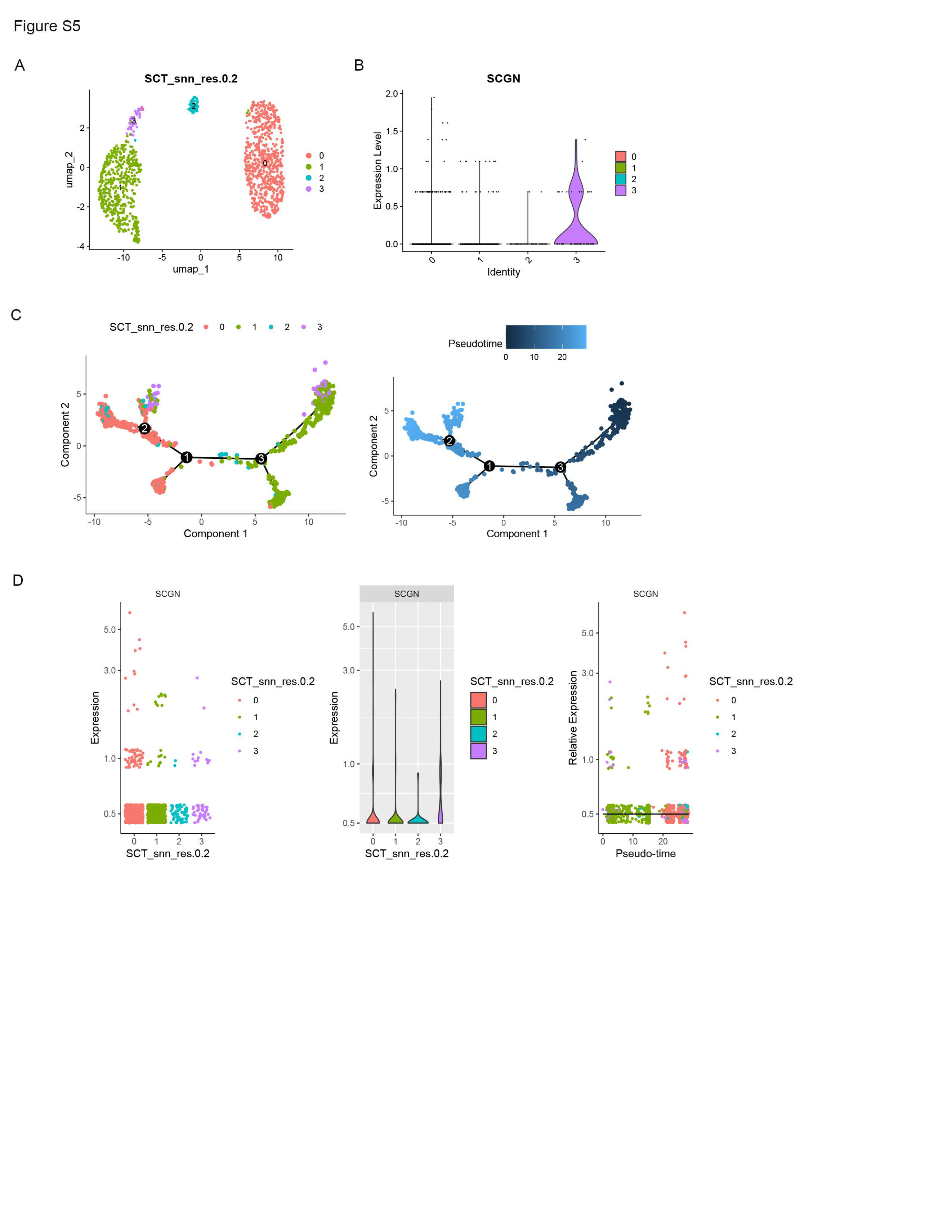

Supplement: Supplementary file 15 [file Image5.tif]
